# TNKS1BP1 facilitates ubiquitination of CNOT4 by TRIM21 to promote hepatocellular carcinoma progression and immune evasion

**DOI:** 10.1038/s41419-024-06897-y

**Published:** 2024-07-17

**Authors:** Yuan Wang, Ineza Karambizi Sandrine, Li Ma, Kailang Chen, Xinyi Chen, Yulong Yu, Sheng Wang, Lingyan Xiao, Chunya Li, Yuanhui Liu, Bo Liu, Xianglin Yuan

**Affiliations:** Department of Oncology, Tongji Hospital, Tongji Medical College, Huazhong University of Science and Technology, Wuhan, 430030 China

**Keywords:** Cancer microenvironment, Autophagy, Fatty acids, Ubiquitylation, Immune evasion

## Abstract

Immune checkpoint inhibitors, particularly PD-1/PD-L1 blockades, have been approved for unresectable hepatocellular carcinoma (HCC). However, high resistance rates still limit their efficacy, highlighting the urgent need to understand the underlying mechanisms and develop strategies for overcoming the resistance. In this study, tankyrasel binding protein 1 (TNKS1BP1) was found to interact with tripartite motif containing 21 (TRIM21) and mediated the ubiquitination of CCR4-NOT transcription complex subunit 4 (CNOT4) at the K239 residue via K48 and K6 linkage, which was essential for its tumorigenesis function. Autophagy and lipid reprogramming were identified as two possible mechanisms underlying the pro-tumor effect of TNKS1BP1. Upregulated TNKS1BP1 inhibited autophagy while induced lipid accumulation by inhibiting the JAK2/STAT3 pathway upon the degradation of CNOT4 in HCC. Importantly, knocking down TNKS1BP1 synergized with anti-PD-L1 treatment by upregulating PD-L1 expression on tumor cells via the JAK2/STAT3 pathway, and remodeling the tumor microenvironment by increasing infiltration of tumor-infiltrating lymphocytes as well as augmenting the effect of cytotoxic T lymphocytes. In conclusion, this study identified TNKS1BP1 as a predictive biomarker for patient prognosis and a promising therapeutic target to overcome anti-PD-L1 resistance in HCC.

## Introduction

Primary liver cancer has become the sixth most commonly diagnosed cancer and the third leading cause of cancer death worldwide. Hepatocellular carcinoma (HCC) comprises 75–80% of liver cancer cases [1]. Prolonged patient survival in the past few years could be attributed to the transition of the therapy from tyrosine kinase inhibitors to immune checkpoint inhibitors [2]. However, no reliable biomarkers predicting response to immune checkpoint inhibitors have been identified in HCC patients yet [3]. Thus, it is of great significance to study the molecular mechanisms governing immune responses and evasion in HCC.

Autophagy is a fundamental mechanism for the maintenance of cellular and organismal homeostasis in response to multiple endogenous and exogenous sources of stress [4]. It plays a double-edged sword role in the biology of cancer [5]. Autophagy plays an inhibition role in the early stage of tumorigenesis, but in the late stage of cancer, autophagy provides energy for cancer cell metabolism to facilitate tumor growth [6]. Autophagy could potentially regulate nearly all aspects of central carbon metabolism including lipid metabolism [7]. Increased de novo fatty acid (FA) and cholesterol biosynthesis is associated with both primary tumor growth and metastasis [8]. Besides, manipulating cholesterol metabolism could also reshape the tumor microenvironment (TME) and alter the efficiency of anti-cancer therapies [9].

Posttranslational modifications refer to amino acid side chain modifications after protein biosynthesis which regulate protein structures and functions [10]. Phosphorylation, methylation, acetylation, glycosylation, palmitoylation, SUMOylation, and ubiquitination are among the most prevalent and critical posttranslational modifications [11, 12]. The ubiquitin-proteasome axis, which affects nearly all essential functions in tumor cells [13, 14], is crucial for maintaining protein homeostasis [15]. Ubiquitination is controlled by the sequential actions of ubiquitin enzymes including E1 ubiquitin-activating enzyme, E2 ubiquitin-conjugating enzyme, and E3 ubiquitin ligase [16]. Eight types of polyubiquitin linkages have been identified (K6, K11, K27, K29, K33, K48, K63, and Met1) with specific functions.

TNKS1BP1 (tankyrasel binding protein 1), also known as TAB182, was first identified in a yeast two-hybrid analysis in 2002 [17]. TNKS1BP1 is a radiation response protein by regulating DNA homologous recombination in lung cancers [18], and enhancing the radioresistance in esophageal squamous cell carcinoma [19], breast cancer, and HCC [20]. However, studies about the biological function of TNKS1BP1 in tumor progression are limited and controversial. TNKS1BP1 has been reported to promote tumor proliferation, invasion, and metastasis in esophageal squamous cell carcinoma [21] and triple-negative breast cancer [22], but TNKS1BP1 was reported to negatively regulates cell invasion in pancreatic cancers [23]. Besides, the role of TNKS1BP1 in cancer immunotherapy has never been elucidated before.

In this study, we revealed that the expression of TNKS1BP1 was positively correlated with the advance stage and poor prognosis in HCC patients. TNKS1BP1 regulated autophagy and lipid metabolism of HCC. Mechanistically, TNKS1BP1 interacted with TRIM21 to form K48- and K6-linked polyubiquitination chains and mediated CCR4-NOT transcription complex subunit 4 (CNOT4) degradation, which promoted the proliferation and migration of HCC. Besides, TNKS1BP1 could synergize with immunotherapy by regulating the JAK2/STST3/PD-L1 pathway and reprogramming the immunogenic landscape in HCC. Taken together, our study highlighted that targeting TNKS1BP1 might be a potential therapy to effectively attenuate the progression and immune evasion in HCC.

## Materials and methods

### Plasmid construction, lentivirus production, and cell transfection

Lentivirus knocking down TNKS1BP1 and Tnks1bp1, and siRNAs targeting CNOT4 and TRIM21 were purchased from OBiO (Shanghai, China). The sequences of the targets of shRNAs and siRNA oligos are listed in Table S1. Flag-TNKS1BP1 was synthesized by Gene Create (Wuhan, Hubei, China). The full-length and truncated fragments of Myc-CNOT4 were synthesized by Miaoling Biology (Wuhan, Hubei, China). His-TRIM21 was synthesized by RiboBio (Guangzhou, Guangdong, China). Point mutated Myc-CNOT4 plasmids were generated with Mut Express II Fast Mutagenesis Kit V2 (C214; Vazyme, Nanjing, Jiangsu, China) following the manufacturer’s protocols, and the sequences of primers were listed in Table S2. Full-length, point-mutated, and truncated fragments of Flag-TRIM21 and the ubiquitin plasmids were kindly provided by Dr. Liu. The sequences of all the plasmids had already been verified by Sanger sequencing at AuGCT (Beijing, China). Transfection of siRNAs and plasmids was performed with Lipofectamine RNAiMAX (13778075; Thermo Fischer Scientific, Van Allen Way Carlsbad, CA, USA) and Lipofectamine 2000 (11668-019; Invitrogen, Thermo Fischer Scientific) according to the manufacturer’s recommendations, respectively. Lentivirus was transfected with polybrene (10 µg/mL) at the multiplicity of infection of 10, and puromycin (2.5 µg/mL, BL528A; Biosharp) was constantly added for 1 week to screen out the cells with stable gene knockdown. Transfection of the adenovirus HBAD-mRFP-GFP-LC3 (Hanbio, Shanghai, China) was carried out following the manufacturer’s protocols. The autophagic flux was captured after 48 h of infection using laser confocal scanning microscopy.

### Flow cytometry analysis

BODIPY 493/503 (10 μM, HY-W090090; MCE) was used to stain the lipid droplets (LDs) according to the manufacturer’s instructions. BODIPY 581/591 C11 (D3861; Invitrogen, Thermo Fischer Scientific) was used to measure the antioxidant activity in lipid environments. In brief, cells were harvested and incubated with 200 μL 10 μM BODIPY 581/591 C11 for 30 min at 37 °C in dark, followed by flow cytometry detection at 581 nm excitation and 591 nm emission. For the detection of PD-L1 on the cell surface, cells were harvested and incubated with 100 μL APC anti-human CD274 antibody (1:400, 29E.2A3; Biolegend, San Diego, CA, USA) for 30 min at room temperature in dark. Flow cytometry analysis was then performed to measure the fluorescence intensity at the indicated wavelength. For the flow cytometry analysis of tissues, fresh Hepa1-6 tumor tissues were harvested, excised and ground into cell suspension. Then the cell suspension was digested in 3 mL DMEM containing DNase I (2 μg/mL, BS137; Biosharp), collagenase IV (1 mg/mL, BS165; Biosharp), and hyaluronidase (10 μg/mL, BS171; Biosharp) at 37 °C for 40 min. After adding 5 mL PBS (BL302A; Biosharp) containing 1% FBS to terminate digestion, the cell suspension was passed through 80 μm strainer filter to obtain single-cell suspension. After lysing in 2 mL red blood cell lysis buffer (R1010; Solarbio, Beijing, China) for 8 min and stopping cell lysis by adding 5 mL PBS containing 1% FBS, cells were stained with fixable viability dye eFluor 780 (65-0865; Invitrogen, Thermo Fischer Scientific) for 20 min at 4 °C in dark. Afterwards, cells were stained with surface antibodies including Alexa Fluor 488 anti-mouse CD45 (30-F11; Biolegend), APC anti-mouse CD3 (17A2; Biolegend), PE anti-mouse CD4 (RM4-4; Biolegend), APC anti-mouse CD8a (53-6.7; Biolegend), anti-mouse CD279 (29 F.1A12; Biolegend), and APC anti-mouse CD274 (10 F.9G2; Biolegend) for 30 min at 4 °C in dark. In the end, cells were fixed in 2% formaldehyde at 4 °C for 30 min in dark. All of the flow cytometry was carried out using the CytoFLEX LX cytometer (Beckman Coulter, Pasadena, CA, USA) and analysis was performed using FlowJo 10.8.1 (FlowJo LLC, Ashland, OR, USA) software.

### Mice xenograft model

All of the animal experiments were performed in accordance with the Declaration of Helsinki and were approved by the Ethics Committee of Tongji Medical College, Huazhong University of Science and Technology (IACUC Issue No.: TJH-202306047). Female C57BL/6 mice (4–5 weeks old) were purchased from GemPharmatech Company (Nanjing, Jiangsu, China). Mice were randomly divided into different groups (*n* = 10 each group, determined based on previous studies), and then Tnks1bp1 knockdown or control Hepa1-6 cells (1 × 10^7^) were harvested and inoculated subcutaneously into the right axilla of mice. When tumor volumes reached 80-100 mm^3^, mice in the treatment groups received intraperitoneal injection of anti-mouse PD-L1 (A2115; Selleck, Houston, USA) every other day for three times. Mice were sacrificed with CO_2_ inhalation when the tumor volume reached 2000 mm^3^. Following data acquisition and analysis were performed in a blinded manner. Tumor volumes were calculated as follows: 0.5 × (*L* × *W*^2^), where *L* = length, *W* = width.

### Statistical analysis

Each experiment was repeated for at least three times. All of the investigators were blinded to group allocation during data collection and analysis procedure. Data were presented as means ± standard deviation (SD). All statistical analyses were performed using GraphPad Prism software (version 8.0.1, GraphPad Software, La Jolla, CA, USA). Comparisons between two groups were conducted using paired *t*-test or unpaired *t*-test depending on variance differences. Comparisons among multiple groups were performed using one-way analysis of variance (ANOVA) or two-way ANOVA depending on variable numbers. *P* values < 0.05 were considered statistically significant.

## Results

### TNKS1BP1 positively correlates with progression and poor prognosis of HCC

**Fig. 1:**
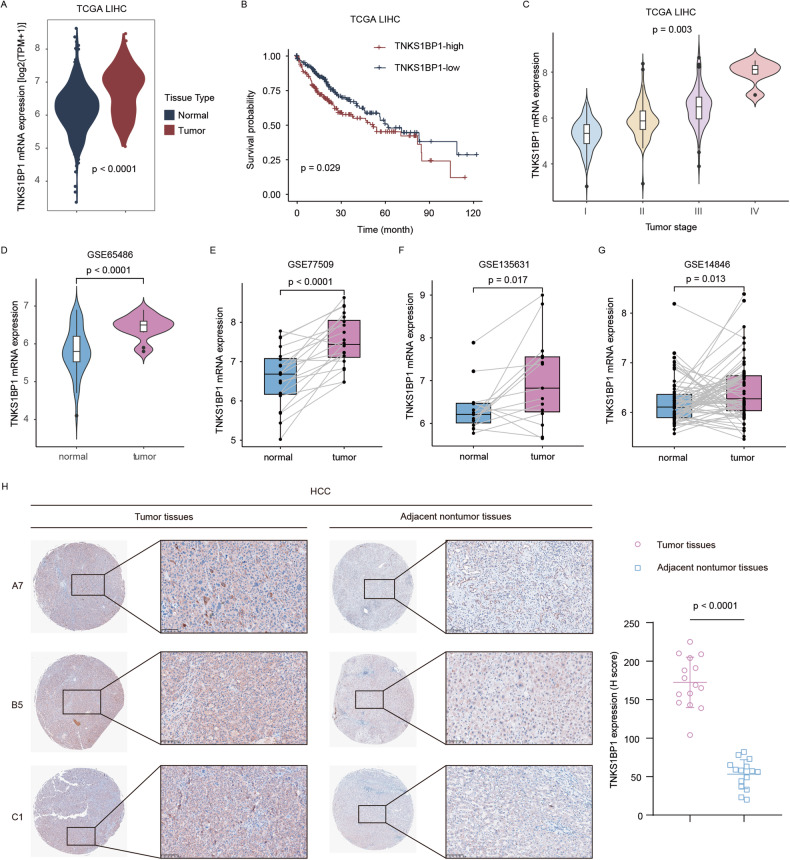
TNKS1BP1 positively correlates with progression and poor prognosis of HCC. **A** Relative mRNA levels of TNKS1BP1 in HCC and normal tissues in the TCGA LIHC cohort. **B** K–M analysis of the OS of HCC patients in the TCGA LIHC cohort based on the expression level of TNKS1BP1. **C** ANOVA analysis of the relevance of TNKS1BP1 expression with tumor stage at diagnosis in the TCGA LIHC cohort. Relative mRNA levels of TNKS1BP1 in HCC and normal tissues in the GSE65486 (**D**), GSE77509 (**E**), GSE135631 (**F**), and GSE14846 (**G**) dataset. **H** Representative images and quantitative analysis of TNKS1BP1 IHC staining in the HCC clinical sample. Scale bars, 100 μm. Significant differences between two groups were analyzed by t-test. Significant differences among multiple groups were analyzed by one-way ANOVA. Error bars represent the means ± SD.

We first aimed to decipher the expression landscape and clinical value of TNKS1BP1 in HCC by analyzing multiple datasets. In the TCGA liver hepatocellular carcinoma (LIHC) dataset, we found that the mRNA levels of TNKS1BP1 were upregulated in human HCC samples compared with normal counterparts (Fig. 1A), and higher expression of TNKS1BP1 was associated with shorter overall survival (OS) in HCC (Fig. 1B). Notably, TNKS1BP1 levels positively correlated with tumor stage at diagnosis (Fig. 1C). Consistent results were found in another four HCC GEO datasets (Fig. 1D–G). Besides, IHC analyses in paired tumor and adjacent nontumorous tissues further confirmed the expression pattern of TNKS1BP1 in HCC (Fig. 1H). Therefore, higher expression of TNKS1BP1 predicted poorer prognosis in HCC patients.

### TNKS1BP1 promotes HCC progression in vitro and in vivo

**Fig. 2:**
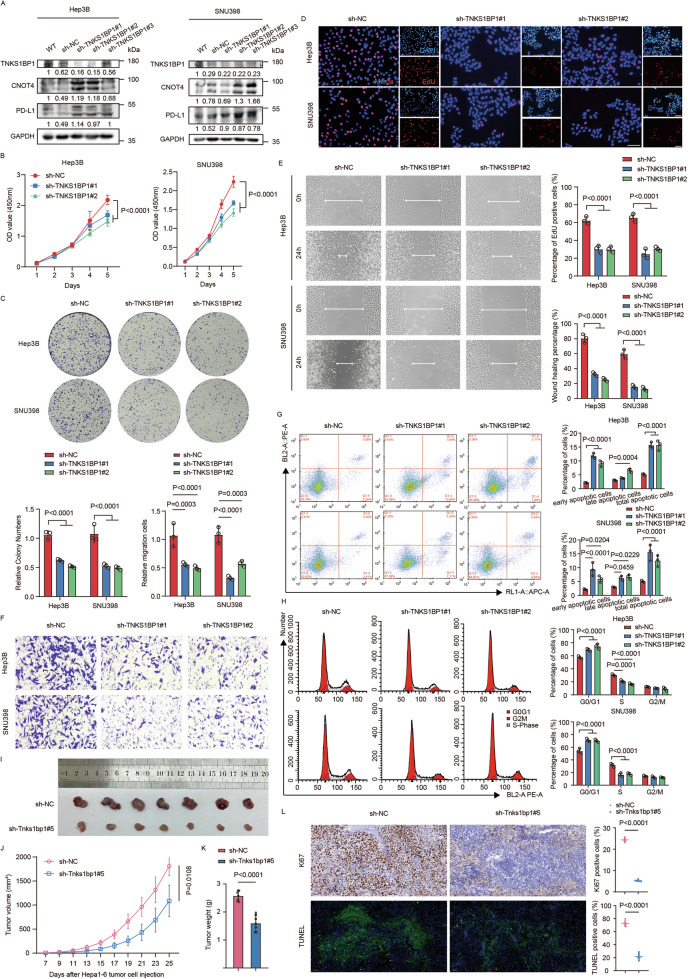
TNKS1BP1 promotes HCC progression in vitro. **A** WB analysis of TNKS1BP1 protein expression levels in TNKS1BP1 knockdown HCC cells. **B** The CCK-8 assay for cell proliferation capacity. Representative images and quantitative analysis (*n* = 3) of the colony formation (**C**), EdU proliferation (**D**), wound healing (**E**), transwell migration (**F**) assay. Scale bars, 100 μm. **G** Representative images and quantitative analysis (*n* = 3) of the flow cytometry analysis detecting the percentage of the early and late stages of apoptotic cells. **H** Representative images and quantitative analysis (*n* = 3) of the flow cytometry analysis detecting the cell phase distribution percentage including G0/G1, S, and G2/M phases. **I** Images of resected subcutaneous tumors. Tumor growth curves (**J**) and tumor weights (**K**) in the subcutaneous tumor model (*n* = 7). **L** Representative images and quantitative analysis of Ki67 IHC staining and TUNEL staining of resected subcutaneous tumors from each group (*n* = 5). Scale bars, 50 μm. Significant differences between two groups were analyzed by *t*-test. Significant differences among multiple groups were analyzed by ANOVA. Error bars represent the means ± SD.

To investigate the biological function of TNKS1BP1, biological effects of TNKS1BP1 knockdown or overexpression on cell proliferation and invasion were assessed. We first measured the endogenous expression of TNKS1BP1 in eight HCC cell lines and found that the expression of TNKS1BP1 was the highest in Hep3B and SNU398 cells, but the lowest in SNU449 cells among all the cell lines at both the transcriptional and translational level (Fig. S1A). Therefore, Hep3B and SNU398 cells were chosen for the construction of stable TNKS1BP1 knockdown cell lines by lentiviral transfection, and SNU449 cells were chosen for the construction of TNKS1BP1 overexpressing cell lines by plasmid transfection (Fig. S1B). The knockdown efficacy of different shRNAs was confirmed by WB and RT-qPCR assays (Fig. 2A; Fig. S1C).

The CCK-8, colony formation and EdU cell proliferation assay indicated that TNKS1BP1 knockdown dramatically inhibited the proliferation of Hep3B and SNU398 cells (Fig. 2B–D). Conversely, ectopic TNKS1BP1 expression promoted cell proliferation (Fig. S1D–F). As shown in the wound healing and transwell migration assay, the migratory ability was also substantially decreased in the TNKS1BP1 knockdown cells (Fig. 2E, F), while the opposite was observed in TNKS1BP1 overexpressing cells (Fig. S1G, H). The flow cytometry assays revealed that TNKS1BP1 knockdown promoted both the early and late stage of apoptosis, as well as the G0/G1 cell cycle arrest in Hep3B and SNU398 cells (Fig. 2G, H). However, the contrary was observed in SNU449 cells overexpressing TNKS1BP1 (Fig. S1I, J). To evaluate the biological function of TNKS1BP1 in vivo, a xenograft tumor model was constructed by inoculating Tnks1bp1 knockdown Hepa1-6 cells subcutaneously into C57BL/6 mice (Fig. S2A). The results confirmed that tumor growth was significantly inhibited after knocking down TNKS1BP1, with no significant impact on the weight of the mice (Fig. 2I–K; Fig. S2B). Moreover, TNKS1BP1 knockdown resulted in proliferation inhibition in xenograft tumors, as indicated by H&E staining and decreased Ki67 and TUNEL levels in xenograft tumor tissues (Fig. 2L; Fig. S2C). Taken together, these findings indicated that TNKS1BP1 promotes HCC growth both in vitro and in vivo.

### TNKS1BP1 regulates autophagy and lipid metabolism reprogramming

To find out the biological process TNKS1BP1 involved in, GSEA was conducted in two GEO datasets which knocked down TNKS1BP1 in triple-negative breast cancer cells (GEO: GSE200038) and HeLa cells (GEO: GSE141496) respectively. The result showed that knocking down TNKS1BP1 significantly activated autophagy while inhibited lipid pathway (Fig. S3A). As autophagy and lipid metabolism are closely related to tumorigenesis, we wanted to figure out the exact role of TNKS1BP1 in these two biological processes.

**Fig. 3:**
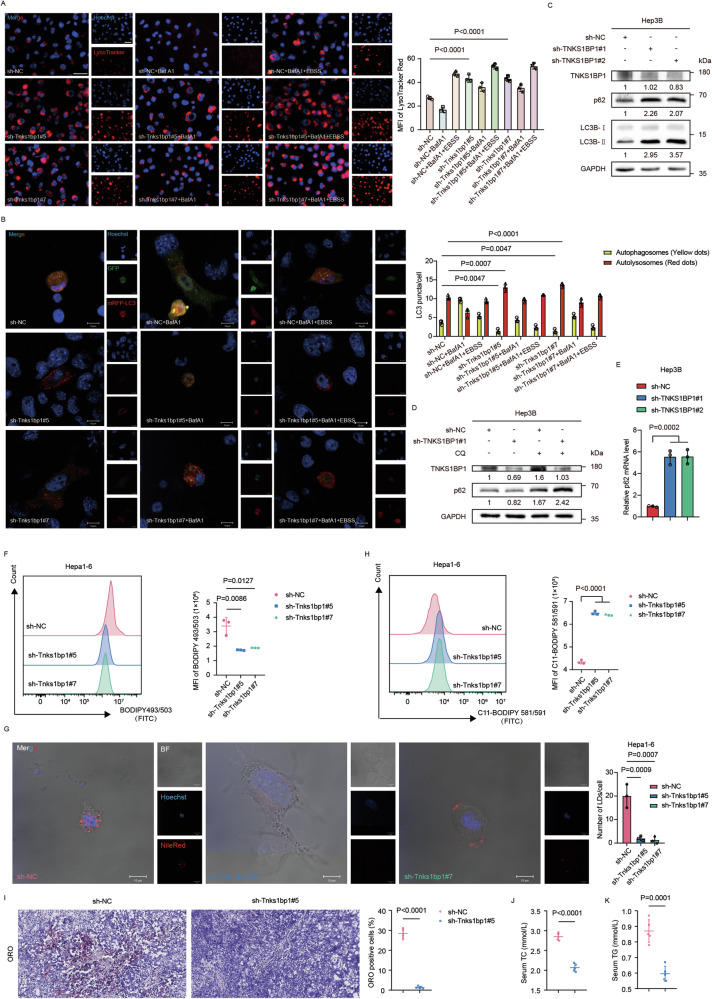
TNKS1BP1 regulates autophagy and lipid metabolism reprogramming. **A** Representative images and MFI of LysoTracker Red staining of lysosomes after treatment with BafA1 and/or EBSS in Tnks1bp1 knockdown and control Hepa1-6 cells (n = 3). Scale bars, 50 μm. **B** Autophagic flux was shown by representative confocal microscope images of Tnks1bp1 knockdown and control Hepa1-6 cells stably expressing GFP-mCherry-LC3 (*n* = 3). Quantitation of autophagosomal (yellow) and autolysosomal (red) LC3 puncta was shown. Scale bars, 10 μm. **C** WB analysis detecting the expression of autophagy-associated protein including LC3B and p62 in TNKS1BP1 knockdown and control Hep3B cells. **D** TNKS1BP1 knockdown and control Hep3B cells were treated with or without CQ and then WB was used to determine the expression of p62. **E** RT-qPCR analysis of p62 mRNA expression levels in TNKS1BP1 knockdown and control Hep3B cells (*n* = 3). **F** The effect of Tnks1bp1 knockdown on lipogenesis in Hepa1-6 cells was determined by BODIPY 581/591 C11 staining followed by flow cytometry analysis (*n* = 3). Representative histograms and MFI were shown. **G** Confocal imaging of Tnks1bp1 knockdown and control Hepa1-6 cells. LDs were stained with Nile Red, and the nucleus were stained with Hoechst 33342. Scale bars, 10 μm. **H** The effect of Tnks1bp1 knockdown on lipid peroxidation in Hepa1-6 cells was determined by BODIPY 493/503 staining followed by flow cytometry analysis (*n* = 3). Representative histograms and MFI were shown. **I** Representative images and quantitative analysis of ORO staining of resected subcutaneous tumors from each group (*n* = 5). Scale bars, 50 μm. Serum TC (**J**) and TG (**K**) level in the subcutaneous tumor model (*n* = 5). Significant differences between two groups were analyzed by t-test. Significant differences among multiple groups were analyzed by ANOVA. Error bars represent the means ± SD.

The autophagic pathway consists of several phases and the degradation of cargoes is dependent on autophagosome-lysosome fusion [24]. To assess the effects of TNKS1BP1 deficiency on lysosome biogenesis, we first evaluated the lysosome numbers using lysosome-specific dye LysoTracker Red in Tnks1bp1 knockdown Hepa1-6 cells with or without the treatment of autophagy inhibitor BafA1 and/or autophagy inducer EBSS. We found that both lysosome numbers and fluorescence intensity were significantly increased after Tnks1bp1 knockdown (Fig. 3A). Then, the autophagic flux of GFP-mCherry-LC3B-labeled Tnks1bp1 knockdown Hepa1-6 cells was evaluated with or without the addition of BafA1 and/or EBSS. As expected, Tnks1bp1 knockdown increased the autophagic flux, as well as autophagosome maturation into autolysosomes in Hepa1-6 cells (Fig. 3B). We further checked the essential proteins involved in the autophagy process by WB and found that TNKS1BP1 knockdown in both Hep3B and SNU398 cells increased the expression of LC3B-II and p62 (Fig. 3C; Fig. S3B). However, it is widely accepted that p62 accumulates in cells when autophagy is inhibited [25], which contradicts our previous conclusions. As a previous study reported that p62 was significantly increased in human HCC tissues [26], to determine whether the increase in p62 protein level was a result of the increased transcription level of p62, the late-stage autophagy inhibitor CQ was added to TNKS1BP1 knockdown HCC cells. CQ could raise the lysosomal pH level and ultimately inhibit the fusion between autophagosomes and lysosomes [27], thus preventing the maturation of autophagosomes into autolysosomes. After CQ treatment, p62 was significantly upregulated in TNKS1BP1 knockdown HCC cells than control cells (Fig. 3D; Fig. S3C). This indicated that autophagy could induce partial p62 degradation. Moreover, the RT-qPCR assay revealed that p62 mRNA level was evidently elevated in TNKS1BP1 knockdown HCC cells (Fig. 3E; Fig. S3D). Taken together, these results confirmed that autophagy was activated after TNKS1BP1 knockdown.

To assess the effect of TNKS1BP1 deficiency on lipid metabolism, we first evaluated intracellular lipid accumulation in Tnks1bp1 knockdown Hepa1-6 cells with the neutral lipid stain BODIPY 493/503 and Nile Red probe. Flow cytometric studies showed that Tnks1bp1 knockdown decreased the BODIPY 493/503 staining in Tnks1bp1 knockdown cells (Fig. 3F). We further examined the abundance of LDs in Hepa1-6 cells with or without Tnks1bp1 knockdown by confocal microscopy. Consistent with the previous results, Nile Red stain also showed lower LDs abundance in Tnks1bp1 knockdown Hepa1-6 cells compared with the control ones (Fig. 3G). Interestingly, we found that Tnks1bp1 knockdown could also increase cellular lipid peroxidation with the BODIPY 581/591 C11 probe (Fig. 3H). De novo fatty acids synthesis (FAS) involves the coordinated actions of several enzymes [28]. We next measured mRNA levels of the key enzymes in the FAS pathway in TNKS1BP1 knockdown and control HCC cells. The transcription levels of multiple enzymes participating in FAS were all significant reduced in TNKS1BP1 knockdown HCC cells (Fig. S3E). Our results were further verified in animal experiments. ORO staining of xenografts indicated that knocking down Tnks1bp1 decreased the accumulation of LDs (Fig. 3I). Besides, serum total cholesterol (TC) level and triglyceride (TG) level were both significantly lower in mice inoculated with Tnks1bp1 knockdown Hepa1-6 cells compared with the control ones (Fig. 3J, K). Altogether, these results indicated that TNKS1BP1 depletion reprogramed lipid metabolism in liver cancer cells.

### TNKS1BP1 interacts with TRIM21 and CNOT4 in HCC cells

**Fig. 4:**
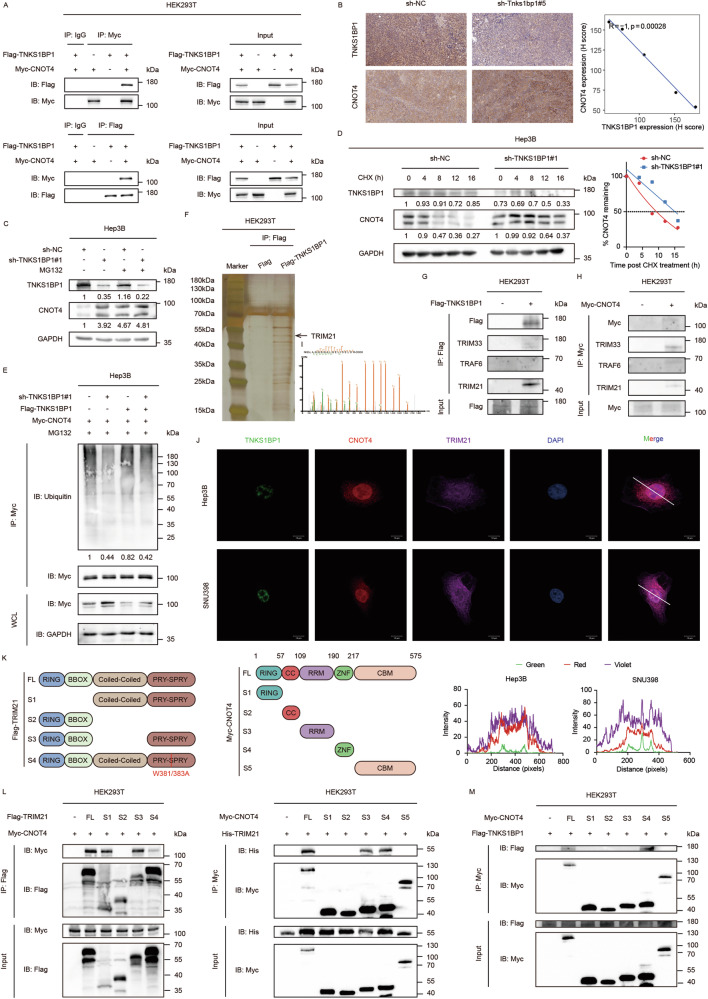
TNKS1BP1 interacts with TRIM21 and CNOT4 in HCC cells. **A** The exogenous interaction between TNKS1BP1 and CNOT4 was determined by performing co-IP and WB assays in HEK293T cells. **B** Representative IHC images of TNKS1BP1 and CNOT4 staining of the subcutaneous tumor model (*n* = 5). Scale bars, 50 μm. The correlation analysis of CNOT4 and TNKS1BP1 staining was shown on the right. **C** WB analysis of CNOT4 level in TNKS1BP1 knockdown and control Hep3B cells stimulated with MG132 (10 μM) for 6 h. **D** The half-life of CNOT4 was determined by CHX (10 μM)-chase assay in TNKS1BP1 knockdown and control Hep3B cells. The CNOT4 band intensities at the indicated time points relative to the initial time point are shown in the graph. **E** The total ubiquitination levels of CNOT4 in TNKS1BP1 knockdown or overexpression Hep3B cells transfected with corresponding plasmids (48 h) and treated with MG132 (10 μM, 6 h). **F** Silver staining of anti-Flag antibody IP products from Flag and Flag-TNKS1BP1 expressing HEK293T cells. The arrow denoted the location of TRIM21. **G** HEK293T cells were transfected with Flag-TNKS1BP1 plasmids. Cell lysates were immunoprecipitated with anti-Flag antibodies and analyzed using immunoblotting. **H** HEK293T cells were transfected with Myc-CNOT4 plasmids. Cell lysates were immunoprecipitated with anti-Myc antibodies and analyzed using immunoblotting. **I** The identified TRIM21 unique peptide sequences. **J** Representative IF images of TNKS1BP1, TRIM21, and CNOT4 colocalization in the nucleus and cytoplasm of Hep3B and SNU398 cells. Scale bars, 10 μm. Line intensity plots showed colocalization of TNKS1BP1, TRIM21, and CNOT4. **K** Schematic of the full-length and truncated mutants of TRIM21 and CNOT4. **L** HEK293T cells were transfected with indicated full-length or truncated mutants of TRIM21 or CNOT4. Cell lysates were collected and subjected to immunoprecipitation with anti-Flag or anti-Myc antibodies to explore the binding regions between TRIM21 and CNOT4. **M** HEK293T cells were transfected with indicated full-length or truncated mutants of CNOT4 and full-length CNOT4. Cell lysates were collected and subjected to immunoprecipitation with anti-Myc antibodies to explore the binding regions between CNOT4 and TNKS1BP1. All the experiments were repeated for at least two times with similar results.

To investigate the mechanisms TNKS1BP1 participated in malignant biological behaviors of HCC cells, we looked for TNKS1BP1 interacting proteins in the GeneMANIA database (https://genemania.org/) [29] and the result showed that CNOT4 had an interaction with TNKS1BP1 (Fig. S4A). This interaction was further validated by co-IP assays in HEK293T cells (Fig. 4A). In addition, we surprisingly found that CNOT4 protein expression level increased in TNKS1BP1 knockdown HCC cells (Fig. 2A), but decreased in TNKS1BP1 overexpressing HCC cells (Fig. S1B). Consistently, IHC analysis of 5 xenograft tumor tissues showed that CNOT4 protein level was negatively correlated with TNKS1BP1 protein level (Fig. 4B). We then performed RT-qPCR assay and found that neither knockdown or overexpression of TNKS1BP1 altered the mRNA level of CNOT4 (Fig. S4B), indicating that TNKS1BP1 regulated CNOT4 mainly through posttranslational modifications. Therefore, we next determined whether TNKS1BP1 induced CNOT4 downregulation in a ubiquitin-proteasome dependent manner. After treatment with the proteasome inhibitor MG132, the difference in CNOT4 protein levels was attenuated in HCC cells (Fig. 4C; Fig. S4C). In addition, we applied CHX to inhibit protein translation and analyzed the effect of TNKS1BP1 on the half-life of CNOT4 protein. TNKS1BP1 knockdown significantly prolonged the half-life of CNOT4 protein in HCC cells (Fig. 4D; Fig. S4D). On the contrary, TNKS1BP1 overexpression significantly shortened the half-life of CNOT4 protein in SNU449 cells (Fig. S4E). The ubiquitination assay showed that TNKS1BP1 knockdown in Hep3B cells obviously attenuated the ubiquitination of CNOT4, while overexpressing TNKS1BP1 significantly increased the ubiquitination level of CNOT4 (Fig. 4E). Taken together, these results indicated that TNKS1BP1 mediated CNOT4 degradation via ubiquitination.

To identify the E3 ubiquitin ligase interacting with TNKS1BP1 that mediates the ubiquitination of CNOT4, a systematic MS analysis following co-IP was conducted to identify TNKS1BP1 interacting proteins by using HEK293T cells stably expressing Flag-TNKS1BP1 (Fig. 4F; Table S4). Three members of the E3 ubiquitin ligase family, including TRIM33, TRAF6 and TRIM21, were identified. Further exogenous reciprocal co-IP assay in HEK293T cells confirmed the interaction between TNKS1BP1 and TRIM21, but not TRIM33 or TRAF6 (Fig. 4G). Interestingly, the exogenous reciprocal co-IP assay in HEK293T cells showed that CNOT4 could interact with all the three E3 ubiquitin ligases (Fig. 4H). Therefore, TRIM21 was chosen as the candidate E3 ligase associated with both TNKS1BP1 and CNOT4 (Fig. 4I). The interaction among TNKS1BP1, CNOT4, and TRIM21 was confirmed by endogenous co-IP assays in HCC cells (Fig. S4F). IF staining followed by confocal analysis also indicated the colocalization of TNKS1BP1, TRIM21, and CNOT4 in the nucleus and cytoplasm of HCC cells (Fig. 4J). To determine the specific binding sequence, a series of TNKS1BP1, TRIM21, and CNOT4 truncation mutants were constructed according to previous studies [23, 30, 31] for co-IP analysis (Fig. 4K). The S4 segment of TRIM21, with tryptophan (W) at the 381 and 383 residues mutating to alanine (A), lost its E3 ubiquitin ligase enzyme activity. The result indicated that the PRYSPRY domain of TRIM21, especially the W381 and W383 residues, was critical for TRIM21 interaction with the RRM and ZNF domain of CNOT4 (Fig. 4L). In addition, the ZNF domain of CNOT4 also interacted with TNKS1BP1 (Fig. 4M). Based on these, we hypothesized that TNKS1BP1 interacted with TRIM21 to facilitate its binding and subsequent degradation of CNOT4. The molecular docking simulations were further used to study the relevant protein-protein interactions (Fig. S4G). Taken together, TNKS1BP1, TRIM21, and CNOT4 had complex protein-protein interactions with each other in HCC cells.

### TRIM21 mediates the K48- and K6-linked ubiquitination of CNOT4 at the K239 residue

**Fig. 5:**
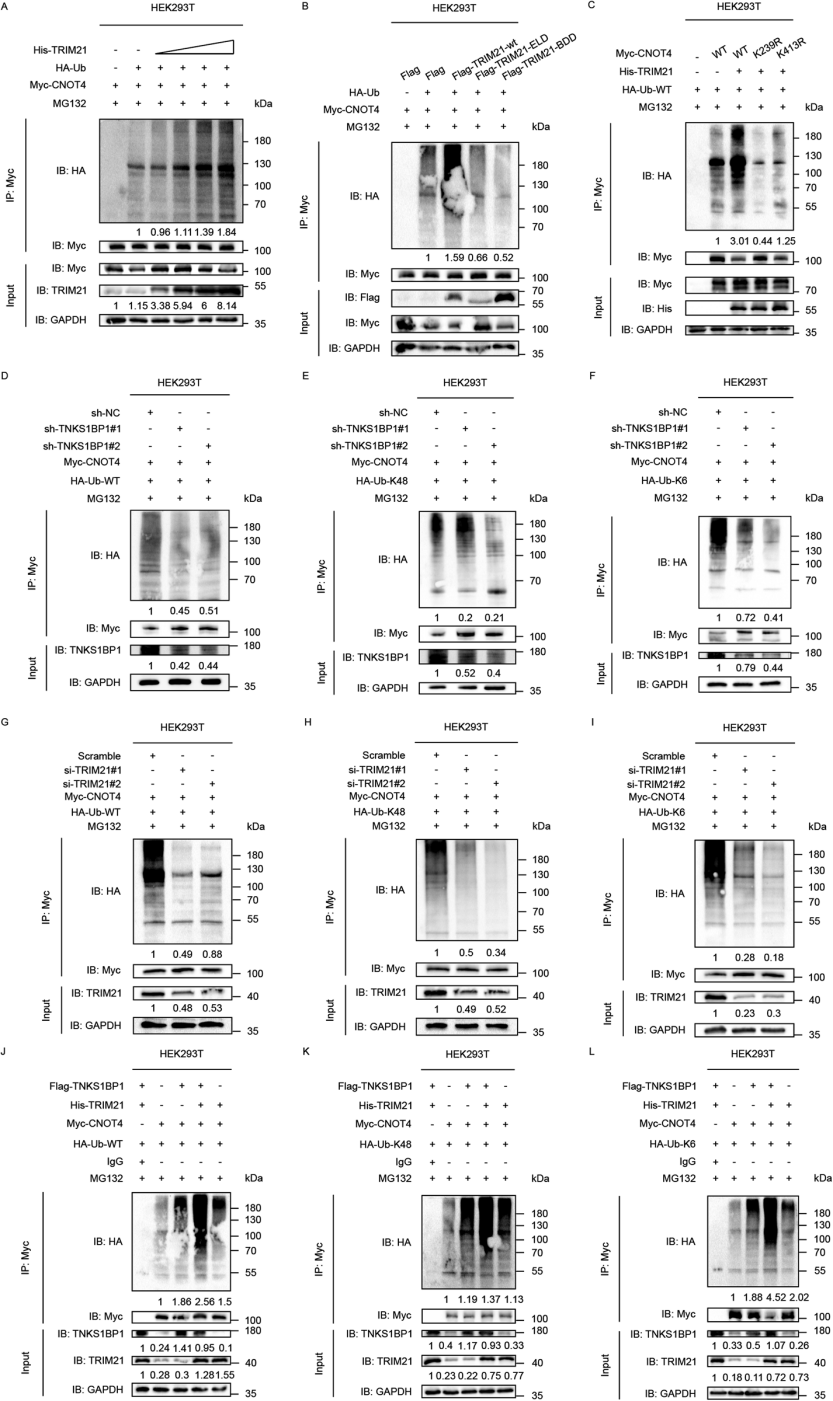
TRIM21 mediates the K48- and K6-linked ubiquitination of CNOT4 at the K239 residue. **A** HEK293T cells were transfected with Myc-CNOT4, HA-Ub, and increasing amounts of His-TRIM21 as indicated. Cell lysates were analyzed by d-IP using anti-Myc antibodies. **B** HEK293T cells were transfected with Myc-CNOT4, HA-Ub, and Flag-tagged wild-type TRIM21 (TRIM21-wt), or TRIM21-ELD, or TRIM21-BDD. Cell lysates were analyzed by d-IP using anti-Myc antibodies. **C** HEK293T cells were transfected with HA-Ub and wild-type or two lysine (K) to arginine (R) mutants of CNOT4. Cell lysates were analyzed by d-IP using anti-Myc antibodies. The total (**D**), K48-linked (**E**), and K6-linked (**F**) ubiquitination levels of CNOT4 in TNKS1BP1 knockdown HEK293T cells transfected with corresponding plasmids (48 h) and treated with MG132 (10 μM, 6 h). The total (**G**), K48-linked (**H**), and K6-linked (**I**) ubiquitination levels of CNOT4 in TRIM21 silenced HEK293T cells transfected with corresponding plasmids (48 h) and treated with MG132 (10 μM, 6 h). The total (**J**), K48-linked (**K**), and K6-linked (**L**) ubiquitination levels of CNOT4 in TNKS1BP1 and TRIM21 overexpressing HEK293T cells transfected with corresponding plasmids (48 h) and treated with MG132 (10 μM, 6 h). All the experiments were repeated for at least two times with similar results.

To verify that TRIM21 could promote CNOT4 ubiquitination, the ubiquitination assay was performed and the result indicated that ectopic expression of TRIM21 increased the ubiquitination level of CNOT4 in a dose-dependent manner (Fig. 5A). In contrast, TRIM21 knockdown increased the protein level of CNOT4 in HCC cells (Fig. S5A). Moreover, both the binding domain-deficient (BDD) mutant (S3) and E3 ligase-deficient (ELD) mutant (S4) and of TRIM21 lost the ability to catalyze the ubiquitination of CNOT4 (Fig. 5B). Next, to identify the critical CNOT4 ubiquitination residues, we predicted the potential ubiquitination site using the online tool PhosphoSitePlus (https://www.phosphosite.org/) [32]. The result predicted K239 and K413 residues, so we mutated lysine (K) at these two residues to arginine (R) using site-directed mutagenesis. The ubiquitination assay showed that the K239R mutation drastically decreased the total ubiquitination level of CNOT4 mediated by TRIM21 (Fig. 5C), indicating that it was an essential ubiquitination site.

It has already been reported that TRIM21 could promote K48-, K63-, K6-, K27-, and K11-linked polyubiquitin chain assembly [33–37]. Therefore, we performed in vivo ubiquitination assays in HEK293T cells and found that knockdown of TNKS1BP1 markedly decreased both the total, K48-, and K6-polyubiquitination, but not K63-, K27, or K11-linked polyubiquitination of Myc-CNOT4 (Fig. 5D–F; Fig. S5B–D). More obvious results were obtained in TRIM21 silenced HEK293T cells (Fig. 5G-I; Fig. S5E–G). Besides, overexpression of both TNKS1BP1 and TRIM21 in HEK293T cells obviously increased the K48- and K6-linked ubiquitination of CNOT4, but not K63-, K27, or K11-linked ubiquitination (Fig. 5J–L; Fig. S5H–J). Collectively, these results suggested that TNKS1BP1 facilitated the K48- and K6-linked ubiquitination of CNOT4 at the K239 residue by TRIM21.

### TNKS1BP1 regulates oncogenesis, autophagy and lipid metabolism of HCC in a CNOT4-dependent manner

Previous studies have already reported that CNOT4 could suppress cancer progression in cancer [38, 39]. Analysis of the TCGA LIHC cohort indicated that CNOT4 expression level was relatively higher in tumor tissues than normal tissues (Fig. S6A). Consistently, K-M survival analysis of the TCGA LIHC cohort revealed that HCC patients with lower CNOT4 expression had a poorer prognosis with lower OS rates compared with patients with higher CNOT4 expression (Fig. S6B).

**Fig. 6:**
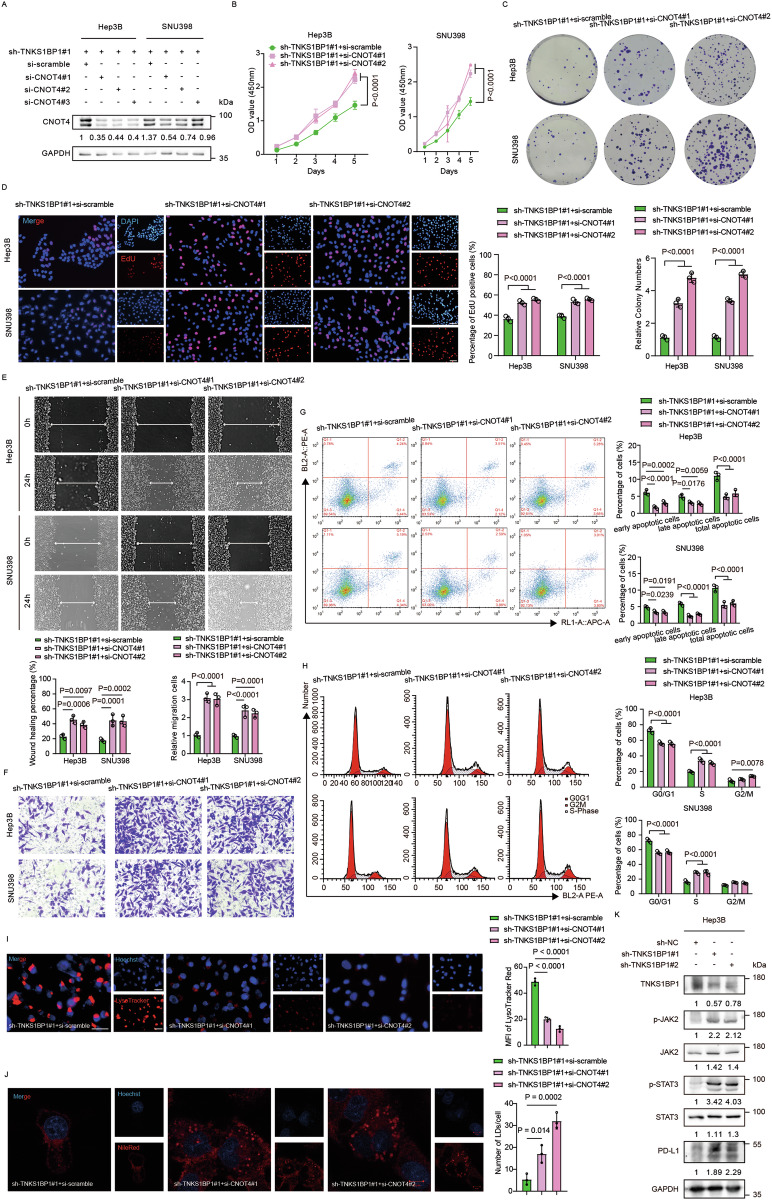
TNKS1BP1 regulates oncogenesis, autophagy, and lipid metabolism of HCC in a CNOT4-dependent manner. **A** WB analysis of CNOT4 protein expression levels in CNOT4 silenced TNKS1BP1 knockdown HCC cells. **B** The CCK-8 assay for cell proliferation capacity. Representative images and quantitative analysis of the colony formation (**C**), EdU proliferation (**D**), wound healing (**E**), and transwell migration (**F**) assay. Scale bars, 100 μm. **G** Representative images and quantitative analysis of the flow cytometry analysis detecting the percentage of the early and late stages of apoptotic cells. **H** Representative images and quantitative analysis of the flow cytometry analysis detecting the cell phase distribution percentage including G0/G1, S, and G2/M phases. **I** Representative images and MFI of LysoTracker Red staining of lysosomes after CNOT4 silencing in TNKS1BP1 knockdown Hep3B cells (*n* = 3). Scale bars, 50 μm. **J** Confocal images of LDs after CNOT4 silencing in TNKS1BP1 knockdown Hep3B cells. Scale bars, 10 μm. **K** WB analysis of p-JAK2, JAK2, p-STAT3, and STAT3 in TNKS1BP1 knockdown and control Hep3B cells. Significant differences among multiple groups were analyzed by ANOVA. Error bars represent the means ± SD of three independent experiments.

We next evaluated whether TNKS1BP1 regulated oncogenesis, autophagy and lipid metabolism in a CNOT4-dependent manner. The transfection efficiency was evaluated by both WB and RT-qPCR assay (Fig. 6A; Fig S6C, D). Consistent with our hypothesis, CNOT4 silencing effectively rescued the inhibition of the proliferation and migration abilities of HCC cells induced by TNKS1BP1 knockdown, as revealed in the CCK-8, colony formation, EdU cell proliferation, wound healing, and transwell migration assays (Fig. 6B–F). Besides, flow cytometry assay showed that CNOT4 silencing reduced both the early and late stages of apoptosis and G0/G1 cell cycle arrest in TNKS1BP1 knockdown HCC cells (Fig. 6G, H). Additionally, CNOT4 overexpression significantly diminished the increased proliferation and migration abilities of TNKS1BP1 overexpressing HCC cells (Fig. S6E–K). Taken together, CNOT4 suppresses the tumor promotion effects of TNKS1BP1 on HCC.

For autophagy, we found that silencing CNOT4 could significantly reduce the increased number and fluorescence intensity of lysosomes induced by TNKS1BP1 knockdown in Hep3B cells (Fig. 6I). Besides, the elevated expression level of LC3B-II and p62 in TNKS1BP1 knockdown Hep3B cells significantly reduced after CNOT4 silencing (Fig. S6L). As for lipid metabolism, CNOT4 silencing effectively rescued the down-regulated transcription level of the enzymes participating in FAS, as well as LDs abundance in TNKS1BP1 knockdown Hep3B cells (Fig. 6J; Fig. S6M). CNOT4 has been identified as a positive regulator of the JAK/STAT pathway [40]. STAT3 was reported to execute its pro-autophagic function by modulating the expression of HIF1A and BNIP3 [41], and it was shown that activation of the JAK2/STAT3 signaling pathway alleviated the accumulation of lipid [42]. Based on these, we wondered whether TNKS1BP1 regulated autophagy and lipid metabolism through the JAK2/STAT3 pathway, so we analyzed the expression level of the main members involved in the pathway. We found that the levels of phosphorylated JAK2 and STAT3 were increased in TNKS1BP1 knockdown HCC cells (Fig. 6K; Fig. S6N). Consistently, the levels of phosphorylated JAK2 and STAT3 were decreased in TNKS1BP1 overexpression SNU449 cells, but increased when CNOT4 was overexpressed at the same time (Fig. S6O). These results indicated that TNKS1BP1 inhibited autophagy and induced lipid accumulation by inhibiting the JAK2/STAT3 pathway.

### TNKS1BP1 inhibition sensitizes HCC to anti-PD-L1 therapy by activating the JAK2/STAT3 pathway and reprograming TME

**Fig. 7:**
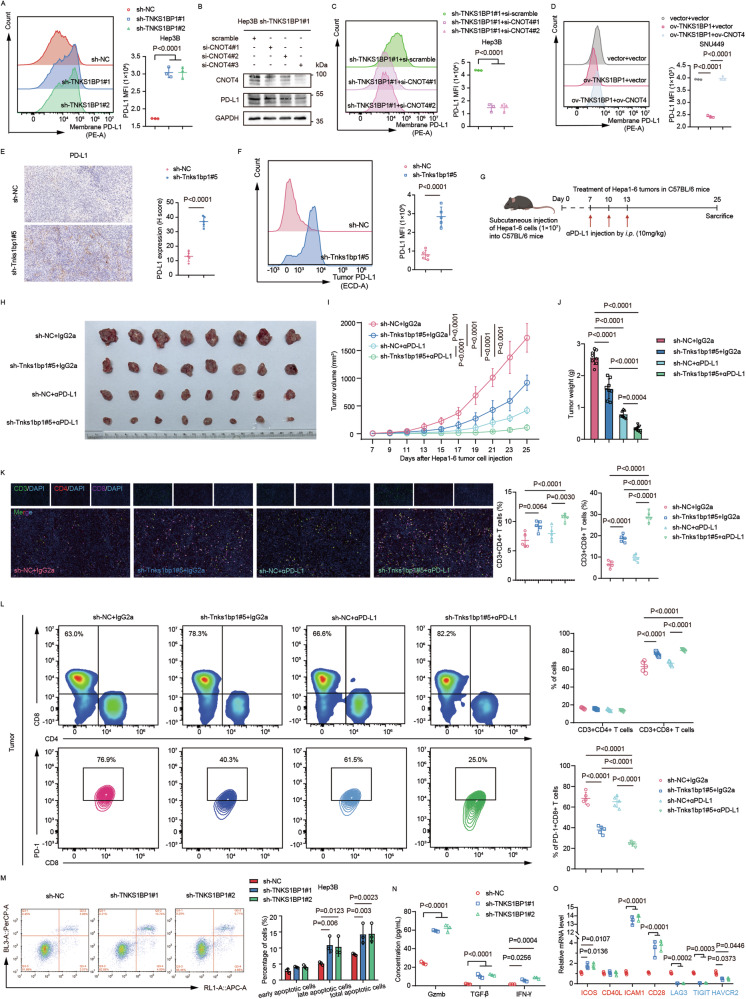
TNKS1BP1 inhibition sensitizes HCC to anti-PD-L1 therapy by activating the JAK2/STAT3 pathway and reprograming TME. **A** Representative images and quantitative analysis of the flow cytometry analysis of cell surface PD-L1 levels in TNKS1BP1 knockdown and control Hep3B cells (*n* = 3). **B** WB analysis of PD-L1 protein expression levels in CNOT4 silenced and control TNKS1BP1-knockdown Hep3B cells. **C** Representative images and quantitative analysis of the flow cytometry analysis of cell surface PD-L1 levels in CNOT4 silenced and control TNKS1BP1-knockdown Hep3B cells (*n* = 3). **D** Representative images and quantitative analysis of the flow cytometry analysis of cell surface PD-L1 levels in the control, TNKS1BP1 overexpression, and TNKS1BP1- and CNOT4-overexpression SNU449 cells (*n* = 3). **E** Representative IHC images of PD-L1 staining of the subcutaneous tumor model (n = 5). Scale bars, 50 μm. **F** Flow cytometry analysis of PD-L1 in xenograft tumor tissues (*n* = 5). **G** Schematic representation of the schedule for anti-PD-L1 therapy in the orthotopic mouse model (n = 8 per group). **H** Images of resected orthotopic tumors. Tumor growth curves (**I**) and tumor weights (**J**) in the orthotopic tumor model (*n* = 8). **K** mIHC analysis and quantification of CD3^+^ CD4^+^ T cells and CD3^+^ CD8^+^ T cells in orthotopic tumor tissues (*n* = 5). Scale bars, 50 μm. **L** The percentage of tumor-infiltrating CD4^+^ T cells, CD8^+^ T cells, and PD-1^+^ CD8^+^ T cells in xenograft tumor tissues (*n* = 5). **M** Representative images and quantitative analysis of the flow cytometry analysis detecting the percentage of apoptotic Hep3B cells (*n* = 5). **N** The level of Gzmb, TGF-β, and IFN-γ were evaluated using ELISA in indicated groups. **O** The level of ICOS, CD40L, ICAM1, CD28, LAG3, TIGIT, and HAVCR2 were evaluated using RT-qPCR in indicated groups. Significant differences between two groups and among multiple groups were analyzed by t-test and ANOVA, respectively. Error bars represent the means ± SD.

Previous results showed that knocking down TNKS1BP1 upregulated CNOT4, which subsequently contributed to the activation of the JAK2/STAT3 pathway. As PD-L1 was regulated by the JAK/STAT pathway [43], and upregulated PD-L1 expression in the TME provides an opportunity for anti-PD-1 and anti-PD-L1 therapy [44], we wondered whether inhibiting TNKS1BP1 could enhance the effect of anti-PD-L1 therapy in HCC by upregulating PD-L1 expression. As expected, we found that PD-L1 protein expression level in the whole cell lysates was significantly higher in TNKS1BP1 knockdown HCC cells than the control ones (Fig. 2A). Knocking down TNKS1BP1 also elevated PD-L1 expression level on the surface of HCC cells (Fig. 7A; Fig. S7A). Silencing CNOT4 in TNKS1BP1 knockdown Hep3B cells could reverse this change in Hep3B cells as detected by WB and flow cytometry (Fig. 7B, C), which was further confirmed by flow cytometry in TNKS1BP1 knockdown SNU398 cells (Fig. S7B). Consistently, flow cytometry detected decreased cell surface PD-L1 expression level in TNKS1BP1 overexpression SNU449 cells, which was reversed when CNOT4 was further overexpressed (Fig. 7D). In vivo experiments verified elevated PD-L1 level in Tnks1bp1 knockdown xenograft tumor tissues as detected by IHC staining and flow cytometry (Fig. 7E, F).

To further explore whether TNKS1BP1 knockdown could sensitize HCC to anti-PD-L1 immunotherapy, we applied bioinformatic analysis and found significant negative correlations between TNKS1BP1 expression level and the infiltration level of most types of T cells, especially CD8^+^ T cells, in the TCGA LIHC cohort (Fig. S7C). To verify this result in vivo, we established orthotopic HCC mouse models bearing Tnks1bp1 knockdown or control cells and treated the mice using an anti-PD-L1 mAb or IgG2a (Fig. 7G). Tumor growth was significantly suppressed by both Tnks1bp1 knockdown and anti-PD-L1 immunotherapy, and combining these two treatments together could achieve the most significant therapeutic effect (Fig. 7H-J). Additionally, no significant difference in the weight of the mice was discovered (Fig. S7D). These results were also verified in H&E, Ki67, and TUNEL staining of xenograft tumor tissues (Fig. S7E). Anti-PD-L1 therapy exerts its antitumor effect mainly by enhancing T cell functions [45]. Therefore, we next mainly focused on T cells to figure out whether knockdown TNKS1BP1 could reshape the TME in the subsequent studies. mIHC staining showed increased infiltration of both CD3^+^ CD4^+^ T cells and CD3^+^ CD8^+^ T cells in the Tnks1bp1 knockdown group, and this phenomenon was even more obvious when combining Tnks1bp1 knockdown and anti-PD-L1 treatment together (Fig. 7K). Flow cytometric analysis of tumor-infiltrating T cells further revealed that apart from increasing total CD8^+^ T cells, combining application of Tnks1bp1 knockdown and anti-PD-L1 treatment could also significantly decrease PD-1^+^ CD8^+^ T cells (Fig. 7L; Fig. S8). We hypothesized that TNKS1BP1 may affect the prognosis of HCC patients by influencing the CD8^+^ T cell-mediated killing effects of tumor cells. To validate this assumption, we co-cultured TNKS1BP1 knockdown and control Hep3B cells with Jurkat T cells. As expected, Hep3B cells with TNKS1BP1 knockdown exhibited higher apoptosis levels compared with the control ones (Fig. 7M). ELISA of the supernatants revealed that CD8^+^ T cell secreted more cytokines including Granzyme B (Gzmb), TGF-β, and IFN-γ when co-cultured with TNKS1BP1 knockdown Hep3B cells compared with the control cells (Fig. 7N). Besides, T cell co-stimulation receptors, including ICOS, CD40L, ICAM1, and CD28, was significantly upregulated on CD8^+^ T cells co-cultured with TNKS1BP1 knockdown Hep3B cells, while T cell co-inhibition receptors, including LAG3, TIGIT, and HAVCR2, was significantly down-regulated (Fig. 7O). Taken together, these results indicated that TNKS1BP1 knockdown could increase the expression level of PD-L1 in tumor cells by activating the JAK2/STAT3 signaling pathway, as well as the infiltration and cytotoxicity of CD8^+^ T cells, which jointly contributed to the increased sensitivity to anti-PD-L1 immunotherapy in HCC patients.

## Discussion

**Fig. 8:**
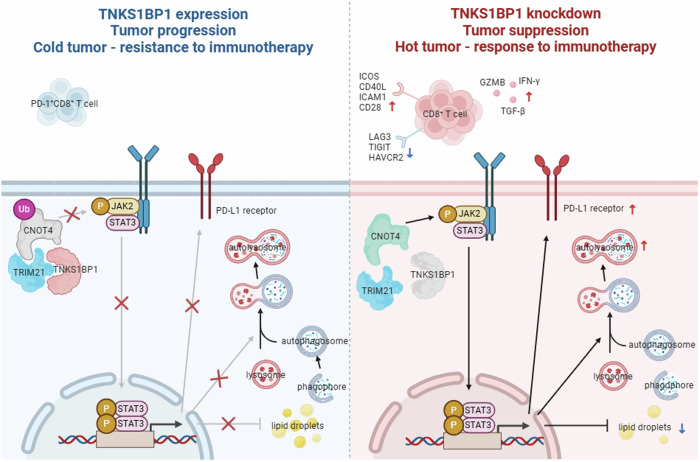
Schematic diagram showing the mechanism by which TNKS1BP1 was involved in tumor progression and antitumor immunity in HCC. Left panel: TNKS1BP1 was abnormally upregulated in HCC cells, which facilitated the ubiquitination and subsequent degradation of CNOT4 by TRIM21, resulting in the inactivation of the JAK2/STAT3 pathway. As a result, LDs synthesis was increased while autophagy was inhibited, leading to tumor progression. More importantly, decreased PD-L1 expression on the cell surface converted the TME of HCC to a “cold” state which was insensitive to immunotherapy. Right panel: knockdown of TNKS1BP1 decreased lipid accumulation, promoted autophagy, and upregulated PD-L1 expression on HCC cell surface by activating the JAK2/STAT3 pathway via CNOT4, resulting in tumor suppression. Targeted inhibition of TNKS1BP1 reshaped the TME by enhancing the recruitment and activation of CD8^+^ T cells, converting the tumor to a “hot” state that was sensitive to immunotherapy. Schematic diagrams were drawn by BioRender (https://www.biorender.com/).

A growing number of clinical trials have already proved the effectiveness of antibodies targeting PD-1/PD-L1 in treating HCC [46–49]. However, the identification of predictive and reliable biomarkers to predict the response to immune checkpoint inhibitors in HCC treatment remains a major challenge. In the present study, we identified a novel biomarker and therapeutic target TNKS1BP1 which was involved in the progress and prognosis of HCC. Briefly, the abnormally upregulated TNKS1BP1 in HCC cells inhibited the autophagy process and promoted the FAS process of HCC cells by deactivating the JAK2/STAT3 pathway, owing to the degradation of CNOT4 via K48- and K6-linked ubiquitination by TRIM21. TNKS1BP1 knockdown abrogated the interaction between CNOT4 and TRIM21, which resulted in increased phosphorylation of JAK2 and STAT3 by CNOT4. Furthermore, TNKS1BP1 knockdown synergized with anti-PD-L1 treatment by upregulating PD-L1 expression on tumor cells via the JAK2/STAT3 pathway and augmenting the infiltration and effector function of cytotoxic T lymphocytes (Fig. 8).

It is widely perceived that FAs are critical for cancer cells as they maintain membrane biosynthesis during rapid cell proliferation and supply an important source of energy under conditions of metabolic stress. In addition, FAs and their by-products have been found to directly regulate intracellular homeostasis, or act as secondary messengers for signal transduction to create the microenvironment favorable for tumor progression [50]. Therefore, suppression of the FAS pathway is a reliable option for tumor therapy. In the present study, we found that TNKS1BP1 knockdown inhibited FA synthesis by inhibiting the key enzymes involved in the process. Whether the transcription of these enzymes was regulated by STAT3 still needs more experiments. Interestingly, we found that TNKS1BP1 knockdown increased cellular lipid peroxidation, which is a hallmark of ferroptosis [51]. Whether TNKS1BP1 could regulate ferroptosis still warrants further exploration. In addition, we only focused on LD synthesis in the current study. Lipolysis and lipophagy are the two pathways of LD degradation [52]. Whether TNKS1BP1 regulates lipophagy also warrants further investigation.

Tripartite Motif-Containing Protein 21 (TRIM21) is an important member of the TRIM family that contains a C-terminal PRYSPRY domain [53], and emerging evidence shows that TRIM21 plays dual roles in cancer [31, 54–57]. In the present study, we found that TRIM21 degraded CNOT4 via K48-linked ubiquitination, which is responsible for the guide of the substrate to the 26S proteasome for degradation [58]. This was consistent with the result that TNKS1BP1 degraded CNOT4 in a ubiquitin-proteasome-dependent manner. Interestingly, TRIM21 also degraded CNOT4 via K6-linked ubiquitination. K6 ubiquitination participates in the process of DNA damage repair [59]. TNKS1BP1 has already been well known as a radiation-responding protein. Whether this three-protein interaction also regulates the DNA damage repair pathway will be investigated in our following experiments.

The TME is considered to play an essential role in tumor initiation, development, metastasis, therapeutic responses as well as clinical outcomes [60]. T cells, mainly consisted of CD4^+^ T helper cells and effector CD8^+^ T cells, are the major tumor-infiltrating lymphocytes in the TME [61]. CD8^+^ T cells play critical roles in eliminating malignant cancer cells and the upregulation of PD-1 on T cells has emerged as a major marker of T cell dysfunction [62, 63]. Activation of CD8^+^ T cells was regulated by a series of co-stimulatory and co-inhibitory molecules on the cell surface of tumor cells and T cells, resulting in the secretion of cytotoxic cytokines [62, 64]. Notably, the expression of PD-L1 is a crucial mechanism in suppressing cytotoxic T lymphocytes (CTLs) function to induce immune tolerance and facilitate tumor escape from the immune system [65]. In the present study, we found that TNKS1BP1 synergized with anti-PD-L1 treatment by increasing infiltration of CTLs, decreasing exhausted CTLs, increasing the expression of co-stimulatory receptors on CTLs, decreasing the expression of co-inhibitory receptors on CTLs, and increasing the secretion of cytotoxic cytokines by CTLs. This was consistent with a previous study which found that HCC patients with increased infiltration of cytotoxic T cells and active circulating CD8^+^ T cells could significantly benefit from pembrolizumab treatment [66]. Besides, cholesterol accumulation in the TME has been shown to increase T cell exhaustion by increasing co-inhibitory surface markers, such as PD-1, TIM-3, and LAG3 [67]. Decreased FAS might partially contribute to the decreased level of exhausted T cells upon TNKS1BP1 knockdown.

In summary, our work provides evidence for the crucial roles of TNKS1BP1 in the progression of HCC and clarifies the TNKS1BP1-TRIM21-CNOT4 axis as a novel proteasome-mediated mechanism. Small molecule inhibitors targeting TNKS1BP1 have the potential to reach a synergistic antitumor efficacy with anti-PD-L1 treatment among HCC patients.

## Supplementary information

Supplementary materials and methods

Supplementary figure legends

Figure S1

Figure S2

Figure S3

Figure S4

Figure S5

Figure S6

Figure S7

Figure S8

Supplementary tables

Original Western Blots

Original Data

## Data Availability

The TGCA LIHC dataset was downloaded from https://xenabrowser.net/. The GEO datasets were downloaded from https://www.ncbi.nlm.nih.gov/geo/. All of the original western blots were provided in Supplementary Data. Other raw data are available upon reasonable request.
