# Correction: TNKS1BP1 facilitates ubiquitination of CNOT4 by TRIM21 to promote hepatocellular carcinoma progression and immune evasion

**DOI:** 10.1038/s41419-024-07183-7

**Published:** 2024-12-19

**Authors:** Yuan Wang, Ineza Karambizi Sandrine, Li Ma, Kailang Chen, Xinyi Chen, Yulong Yu, Sheng Wang, Lingyan Xiao, Chunya Li, Yuanhui Liu, Bo Liu, Xianglin Yuan

**Affiliations:** Department of Oncology, Tongji Hospital, Tongji Medical College, Huazhong University of Science and Technology, Wuhan, 430030 China

**Keywords:** Cancer microenvironment, Autophagy, Fatty acids, Ubiquitylation, Immune evasion

Correction to: *Cell Death and Disease* 10.1038/s41419-024-06897-y, published online 17 July 2024

The original version of this article contained errors in Figure 6. The corrected figure can be found below.
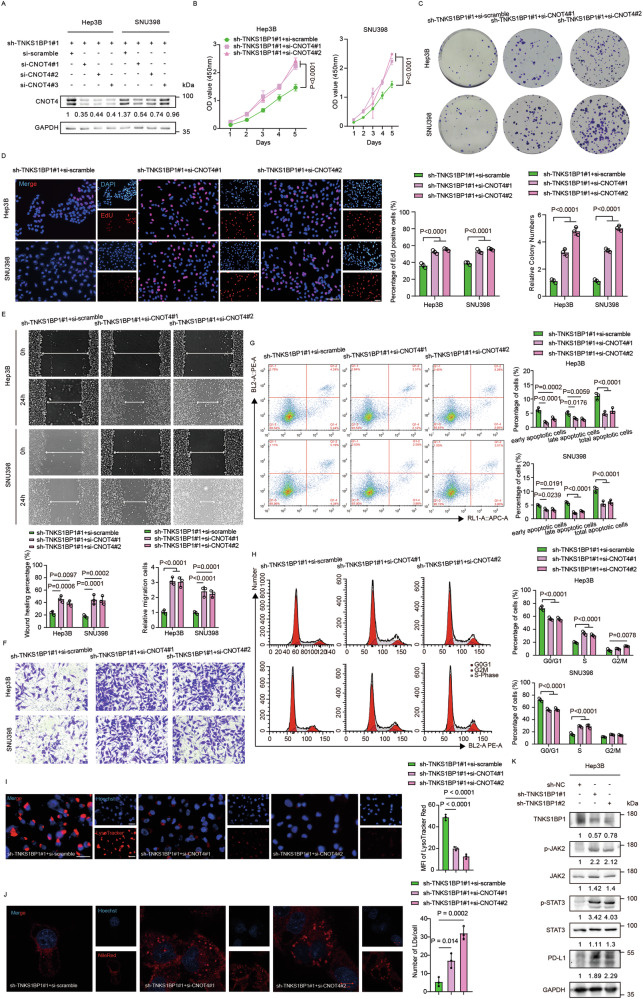


The original article has been corrected.

