# Supplementary material for: TNKS1BP1 facilitates ubiquitination of CNOT4 by TRIM21 to promote hepatocellular carcinoma progression and immune evasion
Source: Cell Death Dis. 2024 Jul 17;15(7):511. doi: 10.1038/s41419-024-06897-y (PMC11255314; doi:10.1038/s41419-024-06897-y)
Supplement: Supplementary file 1 — Supplementary materials and methods [file 41419_2024_6897_MOESM1_ESM.docx]

**Cell lines and cell culture**

HEK293T, HLF, SNU398, SNU449, HepG2, Hep3B, Huh7, MHCC97H, HCCLM3, Hepa1-6, and Jurkat T cells were obtained from the Cell Bank of Chinese Academy of Sciences (Shanghai, China). All cell lines had been authenticated by short tandem repeat (STR) profiling and tested for mycoplasma contamination. All of the adherent cells were cultured in Dulbecco’s Modified Eagle’s Medium (DMEM)/ high glucose (HyClone, Cytiva, Logan, Utah, USA) supplemented with 10% fetal bovine serum (FBS, Newzerum, Christchurch, New Zealand). Jurkat T cells were cultured in Roswell Park Memorial Institute modified medium (HyClone, Cytiva) supplemented with 10% FBS. Cells were cultured in the incubator at 37 ℃ supplemented with 5% CO_2_.

**Antibodies and reagents**

The anti-PD-L1 (13684 & 64988S), anti-LC3B (3868S), and anti-Ki67 (9129) antibodies were obtained from Cell Signaling Technology (CST, Danvers, MA, USA). The anti-Flag antibody (F1804) was obtained from Sigma-Aldrich (Merck, KGaA, Darmstadt, Germany). The anti-Ubiquitin antibody (sc-8017) was obtained from Santa Cruz (Dallas, Texas, USA). The anti-TNKS1BP1 (ab155498), anti-CD3 (ab237721), anti-CD4 (ab183685), anti-CD8 (ab209775), and goat anti-rabbit IgG (ab205718) antibodies was obtained from Abcam (Cambridge, UK). The anti-CNOT4 (67798-1-Ig), anti-TRIM21 (12108-1-AP), anti-TRIM33 (55374-1-AP), anti-His (66005-1-Ig), anti-HA (AE070), anti-TRAF6 (A23385), anti-GAPDH (AC002), anti-p62 (A7758), anti-JAK2 (A19629), anti-STAT3 (A11216), anti-p-JAK2 (AP0531), and anti-p-STAT3 (AP1468) antibodies were purchased from ABclonal (Wuhan, Hubei, China). The peroxidase conjugated goat anti-rabbit IgG (BL003A) and goat anti-mouse IgG (BL001A) antibodies were obtained from Biosharp (Hefei, Anhui, China). Cycloheximide (CHX, HY-12320), chloroquine (CQ, HY-17589A), Bafilomycin A1 (BafA1, HY-100558), and MG132 (HY-13259) were obtained from MedChemExpress (MCE, Monmouth Junction, NJ, USA). Earle's Balanced Salt Solution (EBSS, PWL058) was obtained from MeilunBio (Dalian, Liaoning, China). LysoTracker Red (C1046) and Nile Red (C2051S) were obtained from Beyotime Biotechnology (Wuhan, Hubei, China).

**RNA extraction, reverse transcription, and real-time qPCR (****RT-qPCR) analysis**

Total RNA derived from cells was extracted using RNAiso Plus (9108; Takara Bio, Shiga, Japan) according to the manufacturer’s instructions. RNA was reverse-transcribed into cDNA using HisScript Ⅱ Q RT SuperMix for qPCR (R222; Vazyme). RT-qPCR was performed using ChamQ Universal SYBR qPCR Master Mix (Q711; Vazyme) in Quantagene q225 (Kubotechnology, Beijing, China). Relative gene expression levels were determined with the 2^−ΔΔCT^ method after normalizing to the levels of GAPDH. The sequences of primers used in this study were listed in Table S3.

**Western blot (WB)**

Cells were lysed using RIPA lysis buffer (P0013B; Beyotime Biotechnology) on ice with 1% PMSF (G2008; Servicebio), 1% phosphatase inhibitors (G2007; Servicebio, Wuhan, Hubei, China), and 2% 50 × cocktail protease inhibitors (G2006; Servicebio) for 30 min. After ultrasonication, the lysates were centrifuged at 12 000 rpm for 30 min at 4 ℃. The supernatants were then collected to measure the protein concentration using BCA protein concentration analysis kit (AR0197; Boster, Wuhan, Hubei, China). The supernatants were boiled at 100 ℃ for 5 min after adding 5 × sodium dodecyl-sulfate polyacrylamide gel electrophoresis (SDS-PAGE) sample loading buffer (BL502; Biosharp). The same amount of protein was loaded for SDS-PAGE, followed by transferring to PVDF membranes (IPVH00010; Millipore, Merck KGaA, Darmstadt, Germany). After blocking by 5% non-fat milk at room temperature for 1 h, the membranes were incubated in the indicated primary antibodies at 4 ℃ overnight. After the incubation with the corresponding secondary antibodies at room temperature for 1 h, the signals on the membranes were visualized using SuperSignal West Pico PLUS (34580; Thermo Fischer Scientific) with the G:BOX Chemi X system (Syngene, Cambridge, UK). ImageJ software was used to quantify protein band intensities. Full and uncropped original WB images were displayed in the Original Materials.

**Cell apoptosis, cell cycle, EdU, and cell viability assays**

Cell apoptosis, cell cycle, and EdU assays were performed using the Annexin V-Alexa Fluor647/PI Apoptosis Detection Kit (40304ES20; Yeasen, Shanghai, China), Cell Cycle and Apoptosis Analysis Kit (40301ES60; Yeasen), and Yefluor 594 Edu Imaging Kits (40276ES60; Yeasen) according to the manufacturer’s instructions, respectively. Cell Counting Kit-8 (CCK-8, HY-K0301; MCE) was used to evaluate cell viability measuring absorbance at 450 nm with the microplate reader (BioTek, Winooski, VT, USA) at the indicated time points.

**Colony formation, transwell migration, and wound healing assays**

For the colony formation assay, 500 HCC cells were seeded in six-well plates. Cell colonies were fixed with 4% formaldehyde (BL539A; Biosharp) and stained with crystal violet after 2 weeks. Colonies with more than 50 cells were photographed and quantified. For the transwell migration assay, 5 × 10^4^ HCC cells were inoculated into the upper chambers (TCS003024; Biofil, Guangzhou, Guangdong, China) supplemented with serum-free medium, and the lower chambers were supplemented with complete medium. Migrated cells were fixed with 4% formaldehyde and stained with crystal violet at the indicated time points. For the wound healing assay, HCC cells were seeded into six-well plates and scratched with a 200 μl pipette tip until they reached 100% confluency. The cells were then cultured in serum-free medium and pictures were photographed at the indicated time points. Quantification was completed in ImageJ software.

**In vitro T cell mediated cytotoxicity assay**

Jurkat T cells were activated with PHA-L (2.5 μg/mL, 00-4977-93; Invitrogen, Thermo Fischer Scientific) and PMA (50 ng/mL, HY-18739; MCE) for 24 h. The next day, 1 × 10^5^ HCC cells and 1 × 10^6^ activated Jurkat T cells were seeded in six-well plates. After coculturing for 48 h, HCC cells were harvested to detect the apoptosis rate using the Annexin Ⅴ-APC/7-AAD apoptosis detection kit (KGA1106-20; Keygen Biotech, Nanjing, Jiangsu, China) following the manufacturer’s protocols. Cell supernatants were collected to measure the cytokines secreted by Jurkat T cells using ELISA kits (RK00030, RK00015, and RK00089; ABclonal) according to the manufacturer’s instructions. Jurkat T cells were collected and RNA was extracted to measure the RNA expression levels of T cell co-stimulation and co-inhibition receptors.

**Co-immunoprecipitation (co-IP) and denaturing immunoprecipitation (d-IP)**

For co-IP, cells were collected and lysed in IP lysis buffer (20 mM Tris-HCl at pH 7.4, 1 mM EDTA, 150 mM NaCl, and 1% NP-40, supplemented with protease inhibitors) on ice for 30 min. After centrifugation at 12,000 rpm for 30 min, part of the supernatant was collected and boiled at 100 ℃ for 5 min with 5 × loading buffer as the whole-cell lysate, and the remaining was incubated with the indicated antibodies at 4 °C overnight. The next day, protein A/G magnetic beads (HY-K0202; MCE) were added to the mixture and further incubated at room temperature for 1 h. The beads were then washed three times with PBST and boiled at 95 ℃ with 1 × loading buffer for 5 min. After removing beads, the supernatants were subjected to immunoblot analysis as previously demonstrated. D-IP was used for detecting ubiquitination. In brief, cells were treated with 10 μM MG132 for 6 h before collection. Collected cells were lysed in IP lysis buffer supplemented with 10% SDS solution, and boiled at 95 °C for 5 min. Cell lysates were then diluted tenfold by adding IP lysis buffer and sonicated on ice. The following steps were the same as those described above.

**Silver staining and mass spectrometry (MS) analysis**

After co-IP and fractionation on SDS-PAGE, proteins were stained with fast silver stain kit (BL620A; Biosharp). For LC-MS/MS analysis, the beads samples collected from co-IP were analyzed on the Q Exactive HF mass spectrometer coupled with UltiMate 3000 RSLCnano system at SpecAlly (Wuhan, Hubei, China).

**Immunofluorescence (IF) staining and TUNEL staining**

For multiplex immunohistochemistry (mIHC) of cell samples, cells were first fixed with 4% formaldehyde for 10 min, then 0.1% Triton X-100 was added to permeabilize the cells for 15 min. Then the cell slides were incubated with 10% FBS at 37 ℃ for 30 min. Subsequently, the slices were incubated with the indicated primary antibodies at 4 °C overnight. The next day, the slices were incubated with three secondary antibodies at 37 °C for 45 min. After that, DAPI (1:500, C0060; Solarbio) was added to stain the cell nucleus in dark for 5 min. For mIHC of tissue samples, after desiccation, the paraffin-embedded tissue slices underwent the antigen retrieval process. The following steps were the same as those described above. TUNEL staining was carried out with the TUNEL BrightGreen Apoptosis Detection Kit (A112; Vazyme) according to the manufacturer’s protocols. IF images were captured with Leica DMI3000 B (Leica, Wetzlar, Germany), and the laser confocal scanning was captured on NOL-LSM 710 (Carl Zeiss, Oberkochen, Germany). The fluorescence intensity of each channel was quantified by ImageJ software. Colocalization was determined by the degree of overlap of different curves.

**Oil red O (ORO) staining**

For ORO staining, the fixed frozen samples sections were stained with 60% isopropanol for 20-30 s. Then 40% ORO staining buffer was added to the sections and incubated for 5-10 min. After washing with 60% isopropanol and ddH_2_O, the sections were stained with Mayer hematoxylin for 1 min. Finally, the sections were washed with 1% HCl and water, and sealed for microscope view.

**H&E staining and IHC staining**

For H&E staining, after deparaffinization and rehydration, the paraffin-embedded tissue slices were stained with hematoxylin solution for 4 min and then washed with water for 2 min. Then the slices were differentiated with 0.8% hydrochloric acid alcohol for 2 s and stained with eosin solution for 20 s. After that, the slices were treated with 95% ethanol for 5 min and dehydrated for 2 min. Finally, the tissue sections were made transparent with xylene and mounted with neutral balsam. The expression levels of Ki-67 and PD-L1 proteins in subcutaneous tumors of C57BL/6 mice were evaluated using IHC. In brief, after desiccation, the paraffin-embedded tissue slices underwent the antigen retrieval process. Then endogenous peroxidase was blocked with 3% H_2_O_2_ for 30 min, and incubated with 5% BSA for 30 min. Subsequently, the slices were incubated with primary antibodies against Ki-67 or PD-L1 at 4 °C overnight. The next day, the slices were incubated with HRP-labeled secondary antibodies at 37 °C for 45 min. After applying DAB, hematoxylin was added to stain the cell nucleus for 1 min. Then the slices underwent hydrochloric acid alcohol differentiation followed by blue counterstain.

Images were captured with a histological microscope, and the percentage of positive areas was calculated using ImageJ software. H-score assay was performed to quantify the staining intensities of the protein expression levels by three pathologists independently. Ki-67 and PD-L1 staining was scored according to four categories: 0 for “no staining”, 1^+^ for “light staining”, 2^+^ for “intermediate staining”, and 3^+^ for “dark staining”. The percentage of cells with different staining intensities was determined by visual assessment, with the score calculated using the following formula: 1 × (% of 1^+^ cells) + 2 × (% of 2^+^ cells) + 3 × (% of 3^+^ cells).

**Protein-protein docking**

The 3D structures of TNKS1BP1 (PDB ID: Q9C0C2), CNOT4 (PDB ID: O95628), and TRIM21 (PDB ID: P19474) were obtained from the RCSB database (<https://www.rcsb.org/>). All protein structures were processed in the molecular operating environment (Chemical Computing Group, Montreal, Canada) platform with Amber10 stand selection. Using HDOCK software (Lab of Biophysics and Molecular Modeling, HUST, Wuhan, Hubei, China), the protein was set to rigid, the docking contact site was set to full surface, the conformation generated after docking was set to 100, and the conformation with the most negative energy was selected using the scoring function. Pymol software (DeLano Scientific LLC, South San Francisco, California, USA) was used for visualization. The predicted models and interaction intensities were further analyzed.

**Bioinformatics analysis**

For gene expression and survival analysis, gene expression profiles and clinical information of patients with liver cancer in the Cancer Genome Atlas (TCGA) database and the datasets GSE65486, GSE77509, GSE135631, and GSE14846 were downloaded from the UCSC Xena website (<https://xenabrowser.net/>) and the Gene Expression Omnibus (GEO) website (<https://www.ncbi.nlm.nih.gov/geo/>), respectively. Survival analysis was conducted using the “survival” and “survminer” packages in R software (version 4.3.0) and visualized by Kaplan-Meier (KM) survival curves. For the gene set enrichment analysis (GSEA), differentially expressed genes (DEGs) between the TNKS1BP1 knockdown and control cells were first identified with the R package “limma” in the GEO datasets GSE141496 and GSE200038. Then GSEA was conducted with the R package “clusterProfiler” to determine the signaling pathways in which the DEGs were significantly involved. ESTIMATE algorithm was used to evaluate the absolute abundances of 22 immune cell types in TCGA samples inferred by CIBERSORT (<https://cibersort.stanford.edu/>) ^1^.

**References**

1. Newman AM, Liu CL, Green MR, Gentles AJ, Feng W, Xu Y*, et al.* Robust enumeration of cell subsets from tissue expression profiles. *Nature methods* 2015, **12**(5)**:** 453-457.
