# Supplementary material for: TNKS1BP1 facilitates ubiquitination of CNOT4 by TRIM21 to promote hepatocellular carcinoma progression and immune evasion
Source: Cell Death Dis. 2024 Jul 17;15(7):511. doi: 10.1038/s41419-024-06897-y (PMC11255314; doi:10.1038/s41419-024-06897-y)
Supplement: Supplementary file 2 — Supplementary figure legends [file 41419_2024_6897_MOESM2_ESM.docx]

**Fig. S1 TNKS1BP1 promotes HCC progression in vitro.** **A** WB and RT-qPCR analysis of TNKS1BP1 protein and mRNA expression levels in eight HCC cell lines. **B** WB and RT-qPCR analysis of TNKS1BP1 protein and mRNA expression levels in TNKS1BP1 overexpressing SNU449 cells. **C** RT-qPCR analysis of TNKS1BP1 mRNA expression levels in TNKS1BP1 knockdown HCC cells. **D** The CCK-8 assay for cell proliferation capacity. Representative images and quantitative analysis of the colony formation (**E**), EdU proliferation (**F**), wound healing (**G**), and transwell migration (**H**) assay. **I** Representative images and quantitative analysis of the flow cytometry analysis detecting the percentage of the early and late stages of apoptotic cells. **J** Representative images and quantitative analysis of the flow cytometry analysis detecting the cell phase distribution percentage including G0/G1, S, and G2/M phases. Scale bars, 100 μm. Significant differences between two groups and among multiple groups were analyzed by t-test and ANOVA, respectively. Error bars represent the means ± SD of three independent experiments.

**Fig. S2 TNKS1BP1 promotes HCC progression in vivo. A** WB and RT-qPCR analysis of Tnks1bp1 protein and mRNA expression levels in Tnks1bp1 knockdown and control Hepa1-6 cells (n = 3). **B** Mice weight in the subcutaneous tumor model (n = 7). **C** Representative images of H&E staining of resected subcutaneous tumors from each group (n = 5). Scale bars, 50 μm. Significant differences among multiple groups were analyzed by ANOVA. Error bars represent the means ± SD.

**Fig. S3 TNKS1BP1 regulates autophagy and lipid metabolism reprogramming**. **A** Plots of GSEA between TNKS1BP1 knockdown and control tumor cells in the GEO databases GSE200038 and GSE141496. **B** WB analysis determining the expression of autophagy-associated protein including LC3B and p62 in TNKS1BP1 and control SNU398 cells. **C** TNKS1BP1 and control SNU398 cells were treated with or without CQ and then WB was used to determine the expression of p62. **D** RT-qPCR analysis of p62 mRNA expression levels in TNKS1BP1 knockdown and control SNU398 cells. **E** Relative mRNA expression for genes encoding major enzymes in hepatic lipogenesis in the indicated cells. Significant differences among multiple groups were analyzed by ANOVA. Error bars represent the means ± SD.

**Fig. S4 TNKS1BP1 interacts with TRIM21 and CNOT4 in HCC cells. A** The PPI network of TNKS1BP1-interacting proteins predicted by GeneMANIA. **B** RT-qPCR analysis of CNOT4 mRNA expression levels in TNKS1BP1 knockdown or overexpression HCC cells. **C** WB analysis of CNOT4 level in TNKS1BP1 knockdown and control SNU398 cells stimulated with MG132 (10 μM) for 6 h. **D** The half-life of CNOT4 was determined by CHX (10 μM)-chase assay in TNKS1BP1 knockdown and control SNU398 cells. The CNOT4 band intensities at the indicated time points relative to the initial time point were shown in the graph. **E** The half-life of CNOT4 was determined by CHX (10 μM)-chase assay in TNKS1BP1 overexpression and control SNU449 cells. The CNOT4 band intensities at the indicated time points relative to the initial time point were shown in the graph. **F** The endogenous interaction among TNKS1BP1, TRIM21, and CNOT4 was determined by performing co-IP and WB assays in Hep3B and SNU398 cells. **G** Molecular docking of 3D structures showed the PPIs among TNKS1BP1, TRIM21, and CNOT4. Significant differences between two groups and among multiple groups were analyzed by t-test and ANOVA, respectively. Error bars represent the means ± SD of three independent experiments.

**Fig. S5 TRIM21 mediates the K48- and K6-linked ubiquitination of CNOT4 at the K239 residue. A** WB analysis of TRIM21 and CNOT4 protein levels in TRIM21 silenced Hep3B and SNU398 cells. The K63-linked (**B**), K27-linked (**C**), and K11-linked (**D**) ubiquitination levels of CNOT4 in TNKS1BP1 knockdown HEK293T cells transfected with corresponding plasmids (48 h) and treated with MG132 (10 μM, 6 h). The K63-linked (**E**), K27-linked (**F**), and K11-linked (**G**) ubiquitination levels of CNOT4 in TRIM21-silenced HEK293T cells transfected with corresponding plasmids (48 h) and treated with MG132 (10 μM, 6 h). The K63-linked (**H**), K27-linked (**I**), and K11-linked (**J**) ubiquitination levels of CNOT4 in TNKS1BP1- and TRIM21-overexpressing HEK293T cells transfected with corresponding plasmids (48 h) and treated with MG132 (10 μM, 6 h). All the experiments were repeated for at least two times with similar results.

**Fig. S6 TNKS1BP1 regulates oncogenesis, autophagy and lipid metabolism of HCC in a CNOT4-dependent manner.** **A** The relative mRNA expression level of CNOT4 in normal and tumor tissues from the TCGA LIHC cohort. **B** KM curves showing the OS of HCC patients with high CNOT4 expression level and low CNOT4 expression level in the TCGA LIHC cohort. **C** WB and RT-qPCR analysis of CNOT4 protein and mRNA expression levels in CNOT4 overexpressing TNKS1BP1-overexpressing SNU449 cells. **D** RT-qPCR analysis of CNOT4 mRNA expression levels in CNOT4 silenced TNKS1BP1-knockdown HCC cells. The CCK-8 (**E**), colony formation (**F**), EdU proliferation (**G**), wound healing (**H**), and transwell migration (**I**) assay for cell proliferation capacity. **J** Representative images and quantitative analysis of the flow cytometry analysis detecting the percentage of the early and late stages of apoptotic cells. **K** Representative images and quantitative analysis of the flow cytometry analysis detecting the cell phase distribution percentage including G0/G1, S, and G2/M phases. Scale bars, 100 μm. **L** WB analysis detecting the expression of LC3B and p62 after CNOT4 silencing in TNKS1BP1 knockdown Hep3B cells. **M** Relative mRNA expression of major enzymes participating in the FAS pathway in the indicated cells. **N** WB analysis of p-JAK2, JAK2, p-STAT3, and STAT3 in TNKS1BP1 knockdown and control SNU398 cells. **O** WB analysis of p-JAK2, JAK2, p-STAT3, and STAT3 in the control, TNKS1BP1-overexpression, and TNKS1BP1- and CNOT4-overexpression SNU449 cells.Significant differences between two groups and among multiple groups were analyzed by t-test and ANOVA, respectively. Error bars represent the means ± SD of three independent experiments.

**Fig. S7 TNKS1BP1 inhibition sensitizes HCC to anti-PD-L1 therapy by activating the JAK2/STAT3 pathway and reprograming TME. A** Representative images and quantitative analysis of the flow cytometry analysis of cell surface PD-L1 levels in TNKS1BP1 knockdown and control SNU398 cells (n = 3). **B** Representative images and quantitative analysis of the flow cytometry analysis of cell surface PD-L1 levels in CNOT4 silenced and control TNKS1BP1-knockdown SNU398 cells (n = 3). **C** Spearman correlations between TNKS1BP1 expression level and the absolute abundance of different T cell types estimated by CIBERSORT for the TCGA LIHC cohort. **D** Mice weight in the subcutaneous tumor model (n = 8). **E** Representative images and quantitative analysis of H&E staining, Ki67 IHC staining, and TUNEL staining of resected subcutaneous tumors from each group (n = 5). Scale bars, 50 μm. Significant differences between two groups and among multiple groups were analyzed by t-test and ANOVA, respectively. Error bars represent the means ± SD.

**Fig. S8 Gating and sorting strategies.** Representative flow cytometry gating strategies of CD4^+^ T cells, CD8^+^ T cells, and PD-1^+^CD8^+^ T cells in xenograft tumor tissues of HCC mouse models.
