# Supplementary figures and images for: TNKS1BP1 facilitates ubiquitination of CNOT4 by TRIM21 to promote hepatocellular carcinoma progression and immune evasion
Source: Cell Death Dis. 2024 Jul 17;15(7):511. doi: 10.1038/s41419-024-06897-y (PMC11255314; doi:10.1038/s41419-024-06897-y)

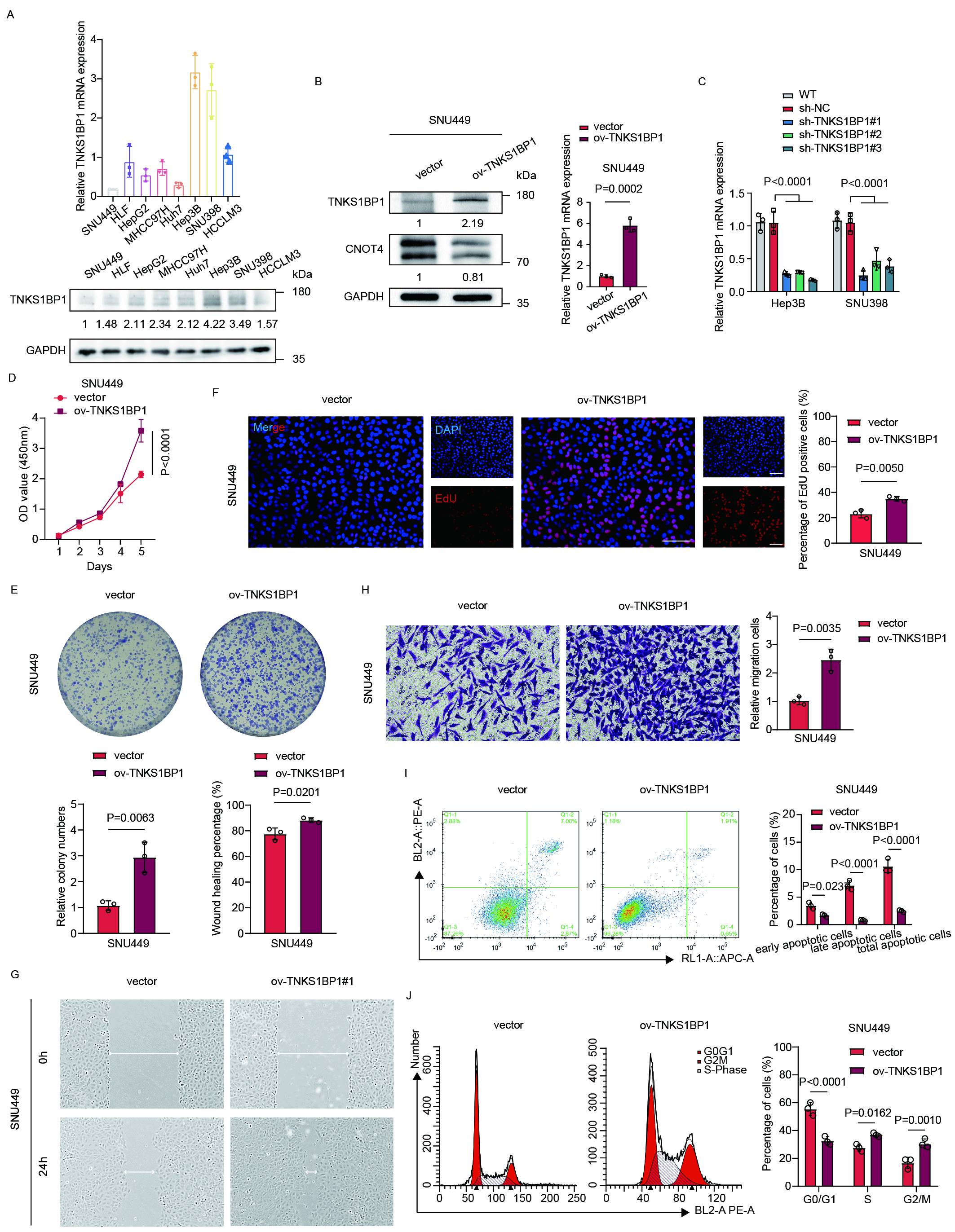

Supplement: Supplementary file 3 — Figure S1 [file 41419_2024_6897_MOESM3_ESM.tif]

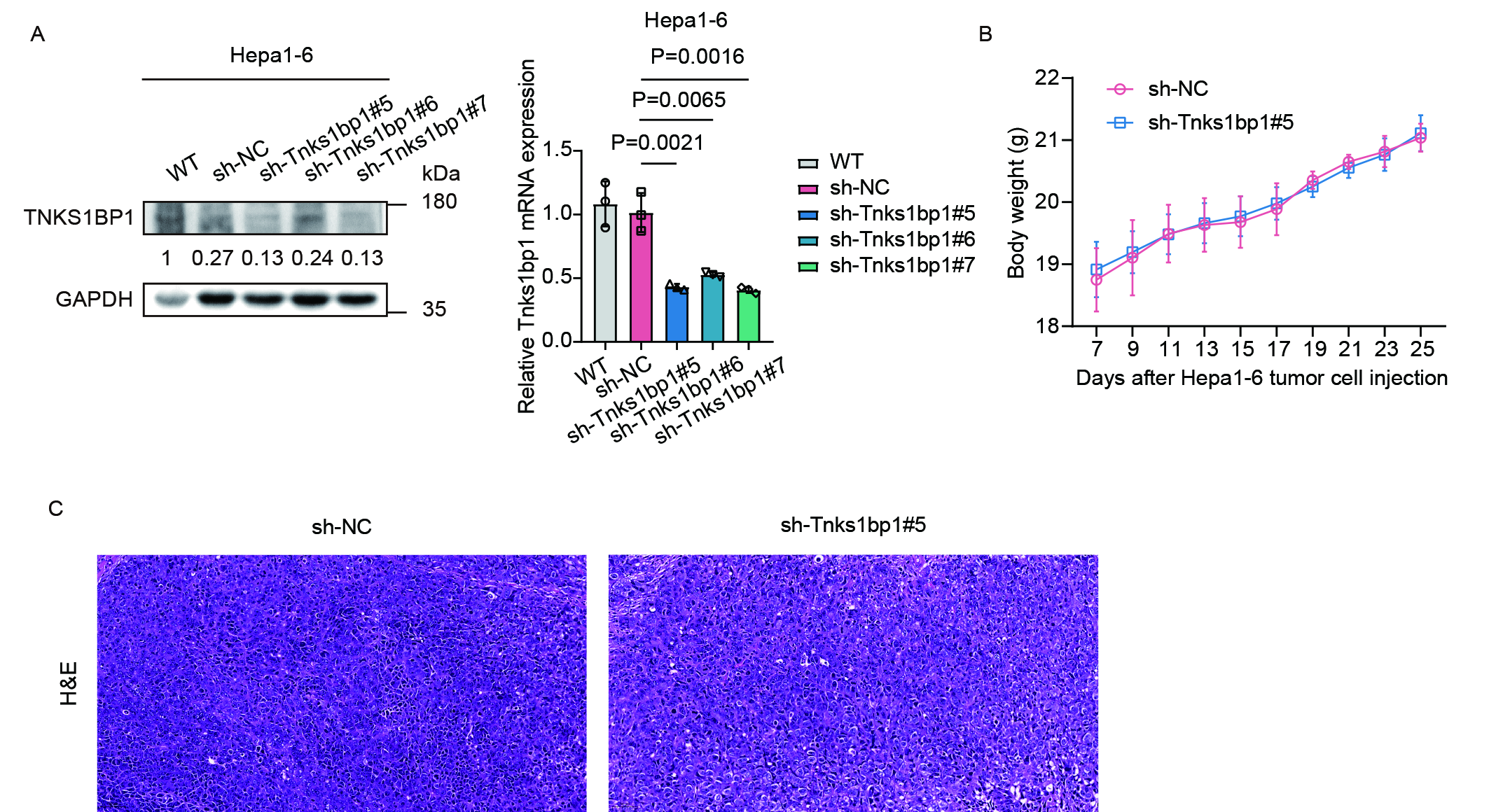

Supplement: Supplementary file 4 — Figure S2 [file 41419_2024_6897_MOESM4_ESM.tif]

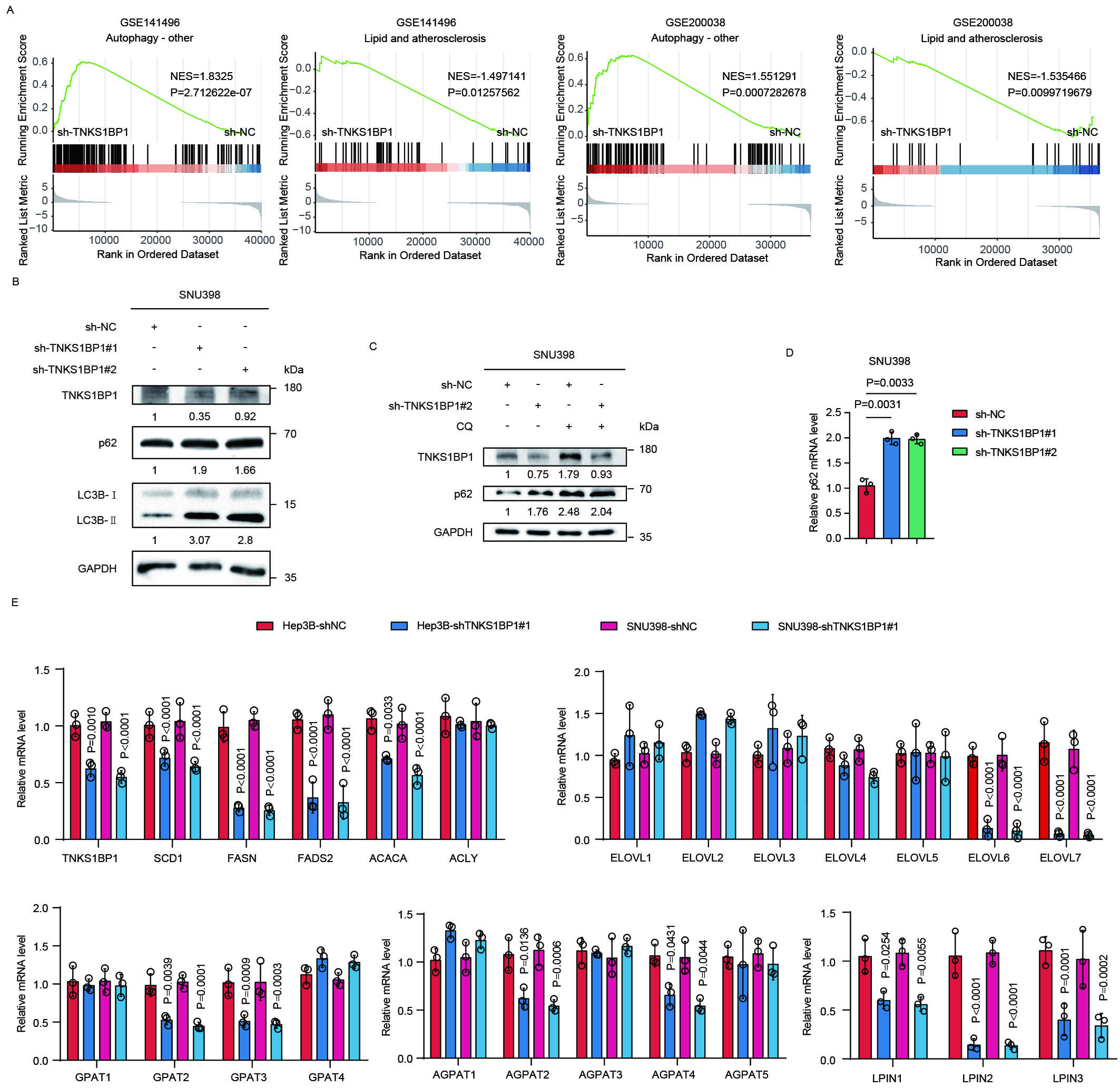

Supplement: Supplementary file 5 — Figure S3 [file 41419_2024_6897_MOESM5_ESM.tif]

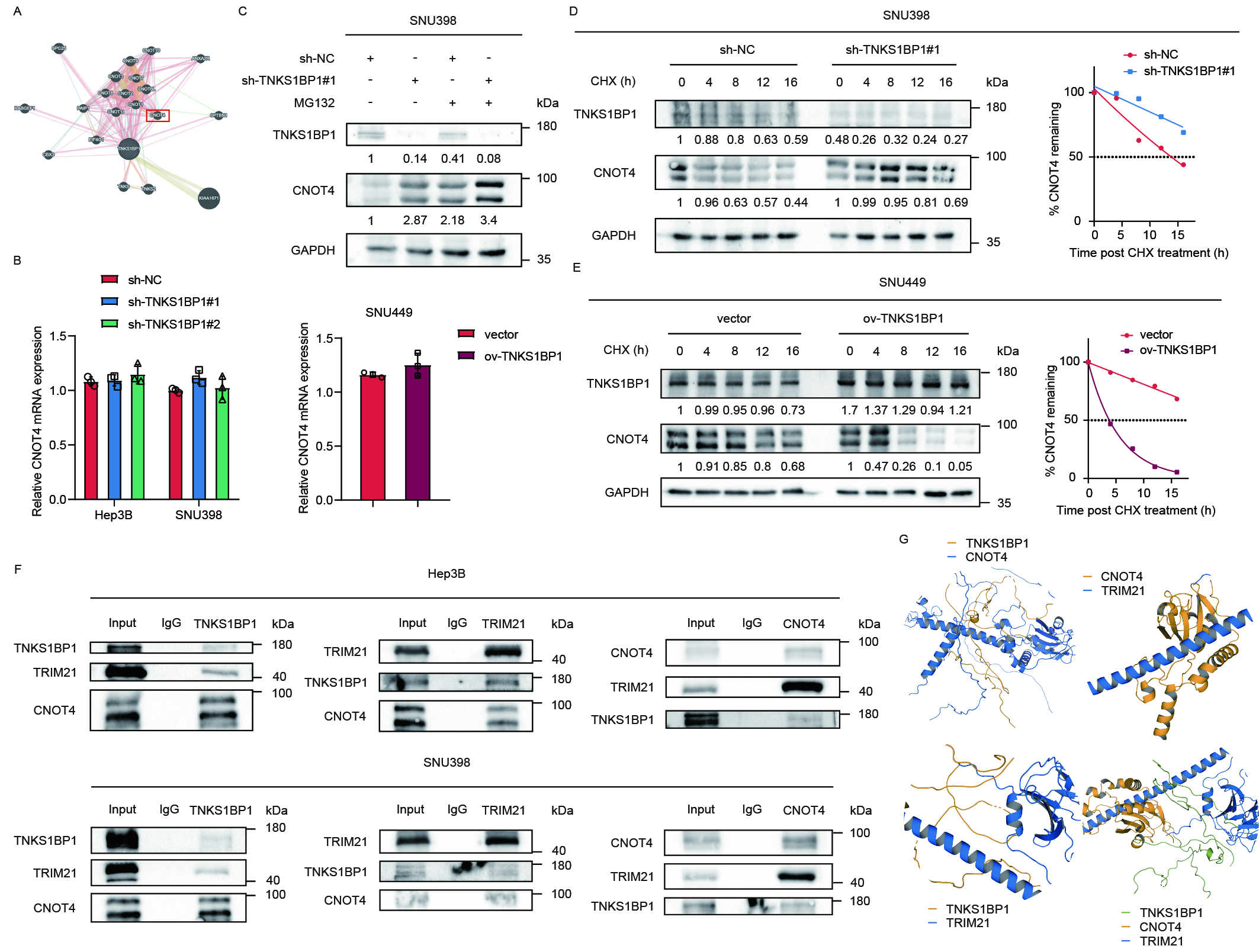

Supplement: Supplementary file 6 — Figure S4 [file 41419_2024_6897_MOESM6_ESM.tif]

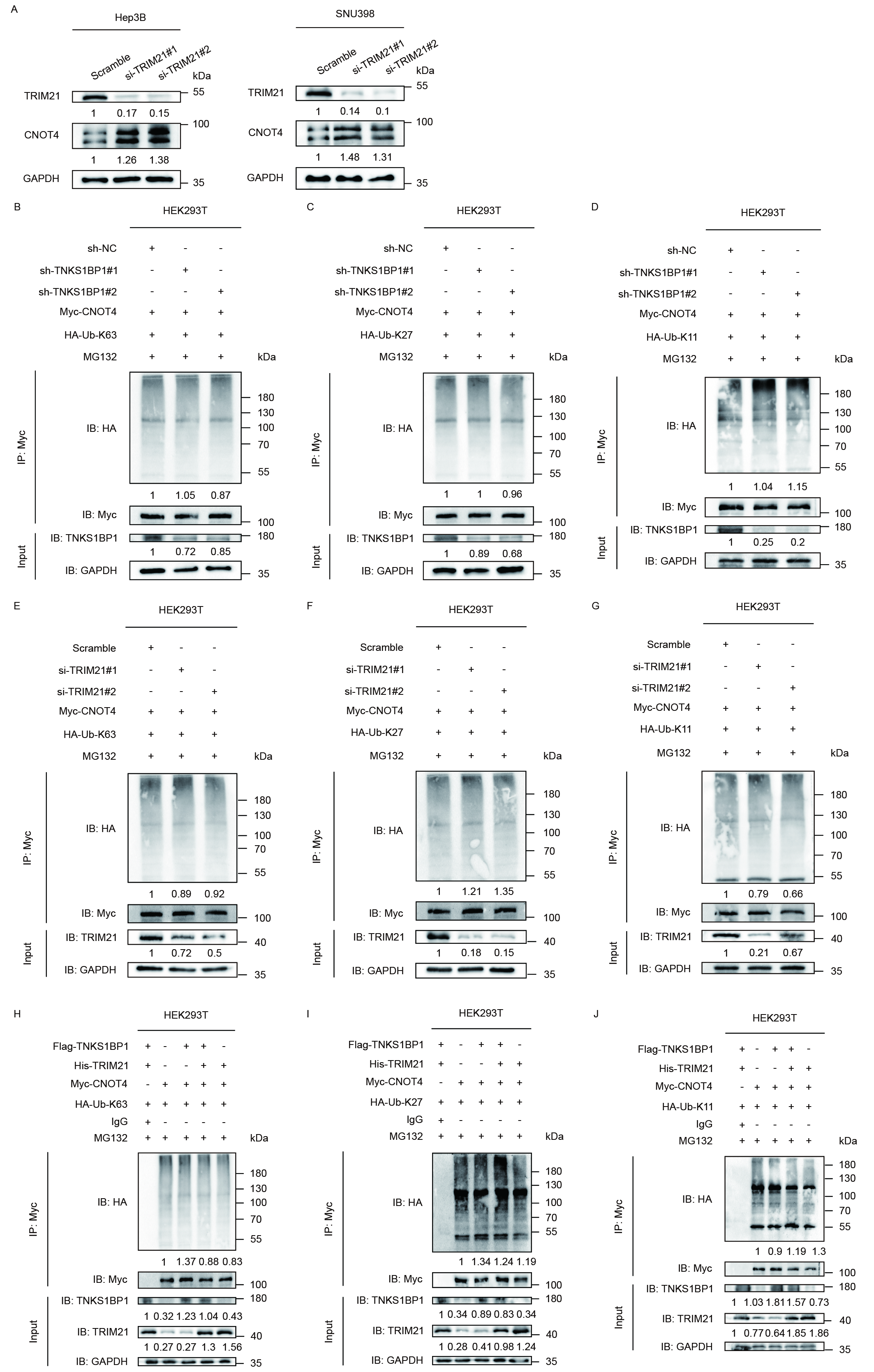

Supplement: Supplementary file 7 — Figure S5 [file 41419_2024_6897_MOESM7_ESM.tif]

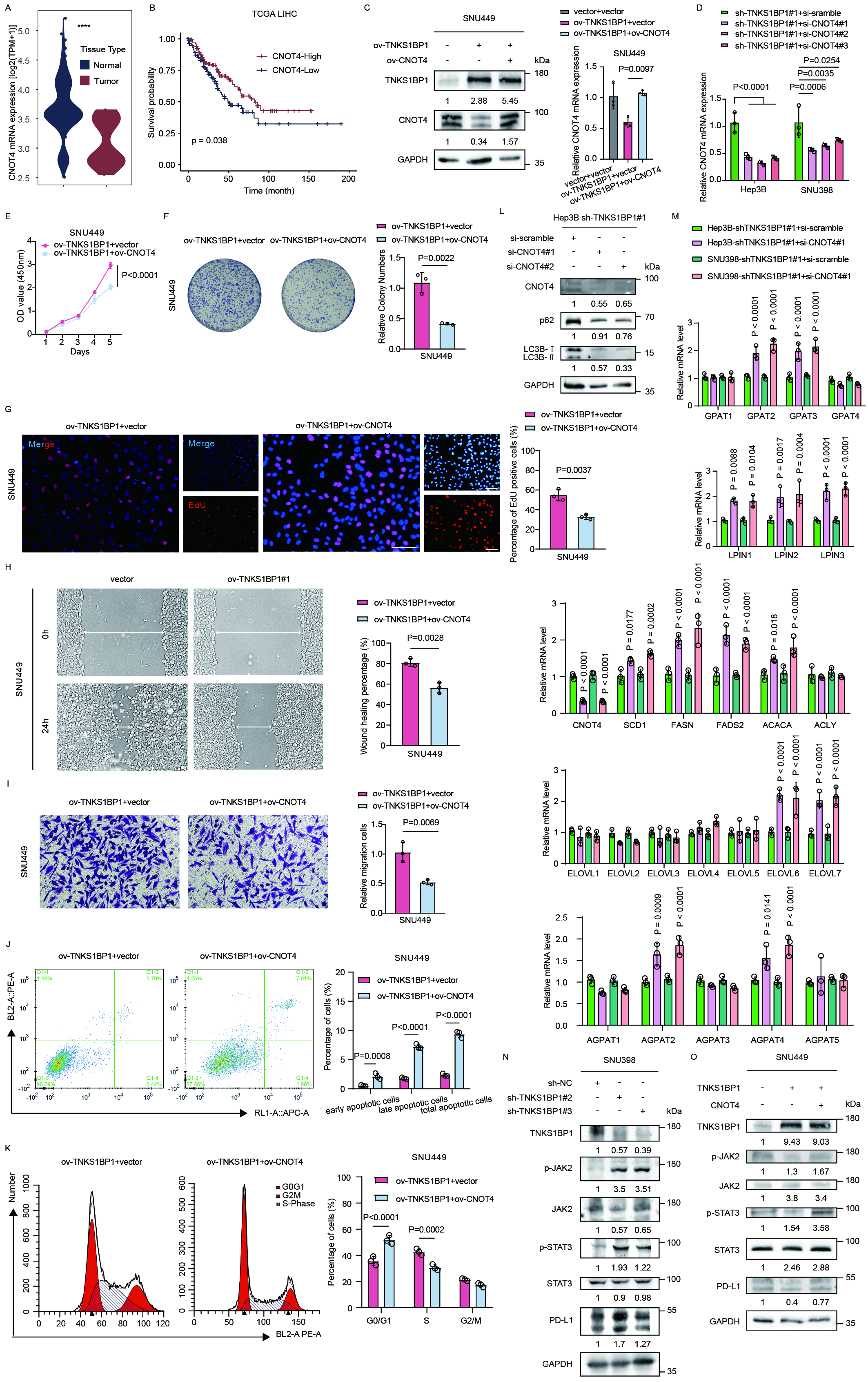

Supplement: Supplementary file 8 — Figure S6 [file 41419_2024_6897_MOESM8_ESM.tif]

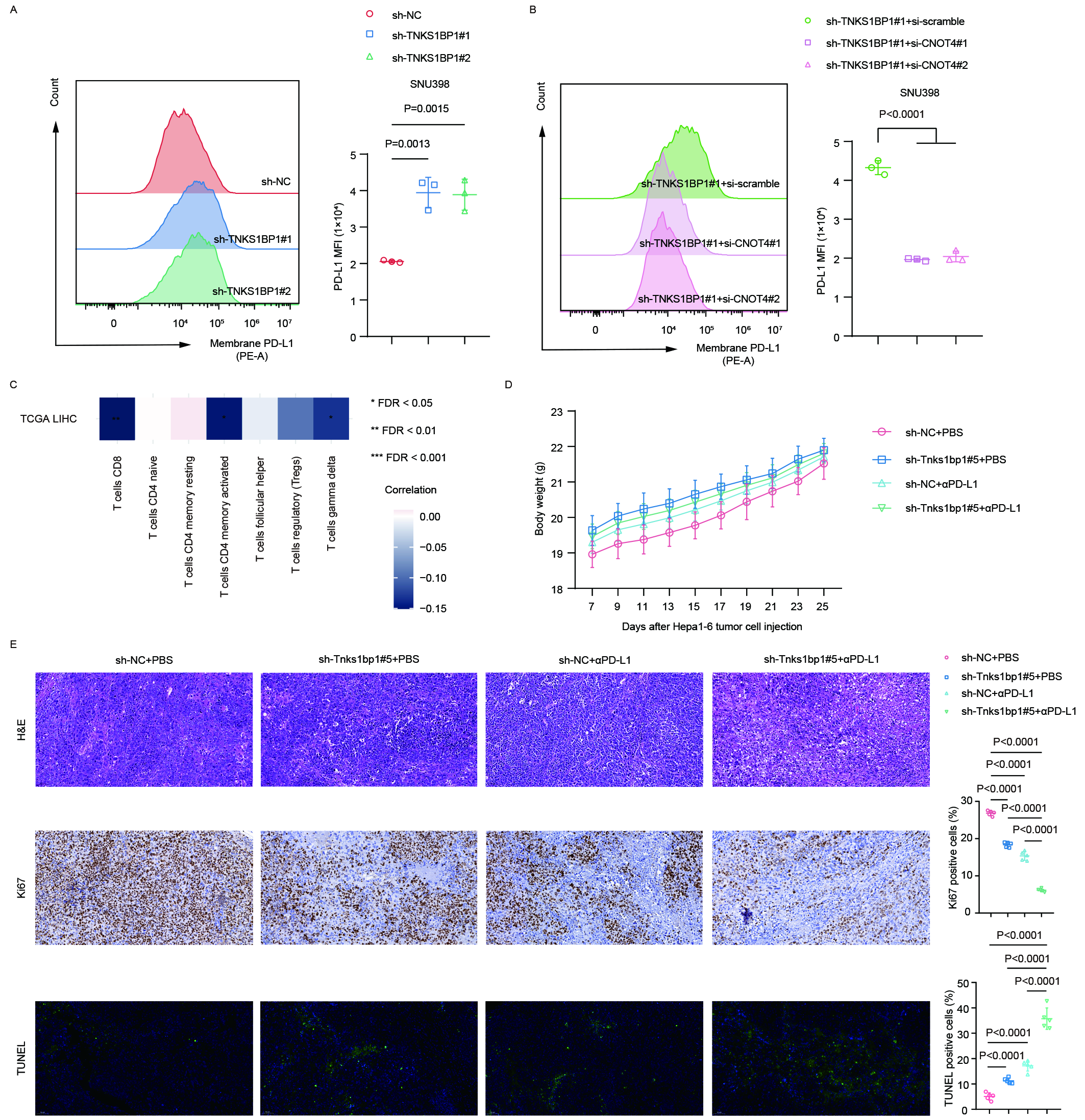

Supplement: Supplementary file 9 — Figure S7 [file 41419_2024_6897_MOESM9_ESM.tif]

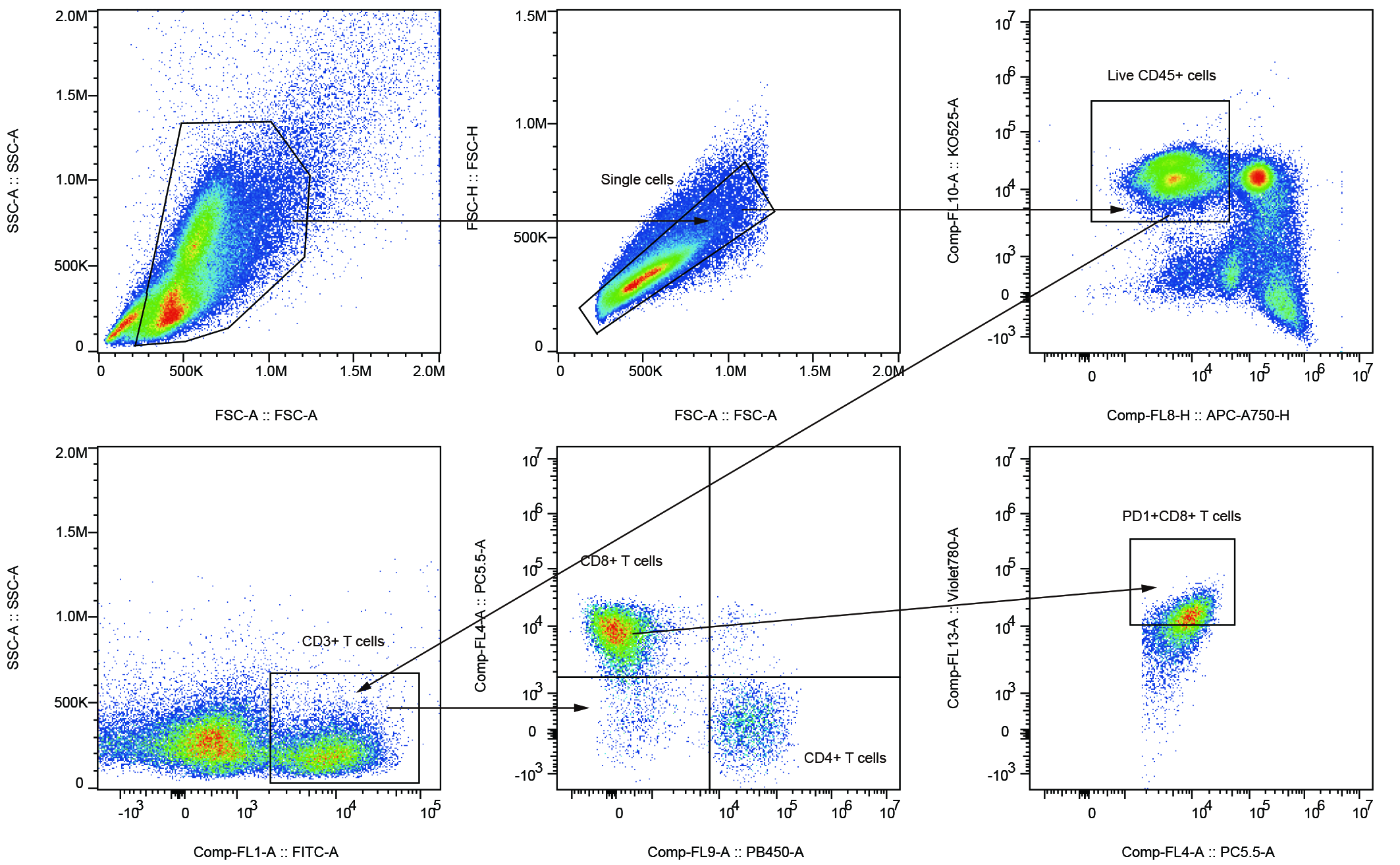

Supplement: Supplementary file 10 — Figure S8 [file 41419_2024_6897_MOESM10_ESM.tif]
