# Supplementary material for: TNKS1BP1 facilitates ubiquitination of CNOT4 by TRIM21 to promote hepatocellular carcinoma progression and immune evasion
Source: Cell Death Dis. 2024 Jul 17;15(7):511. doi: 10.1038/s41419-024-06897-y (PMC11255314; doi:10.1038/s41419-024-06897-y)

Unprocessed western blots for indicated figure panels.

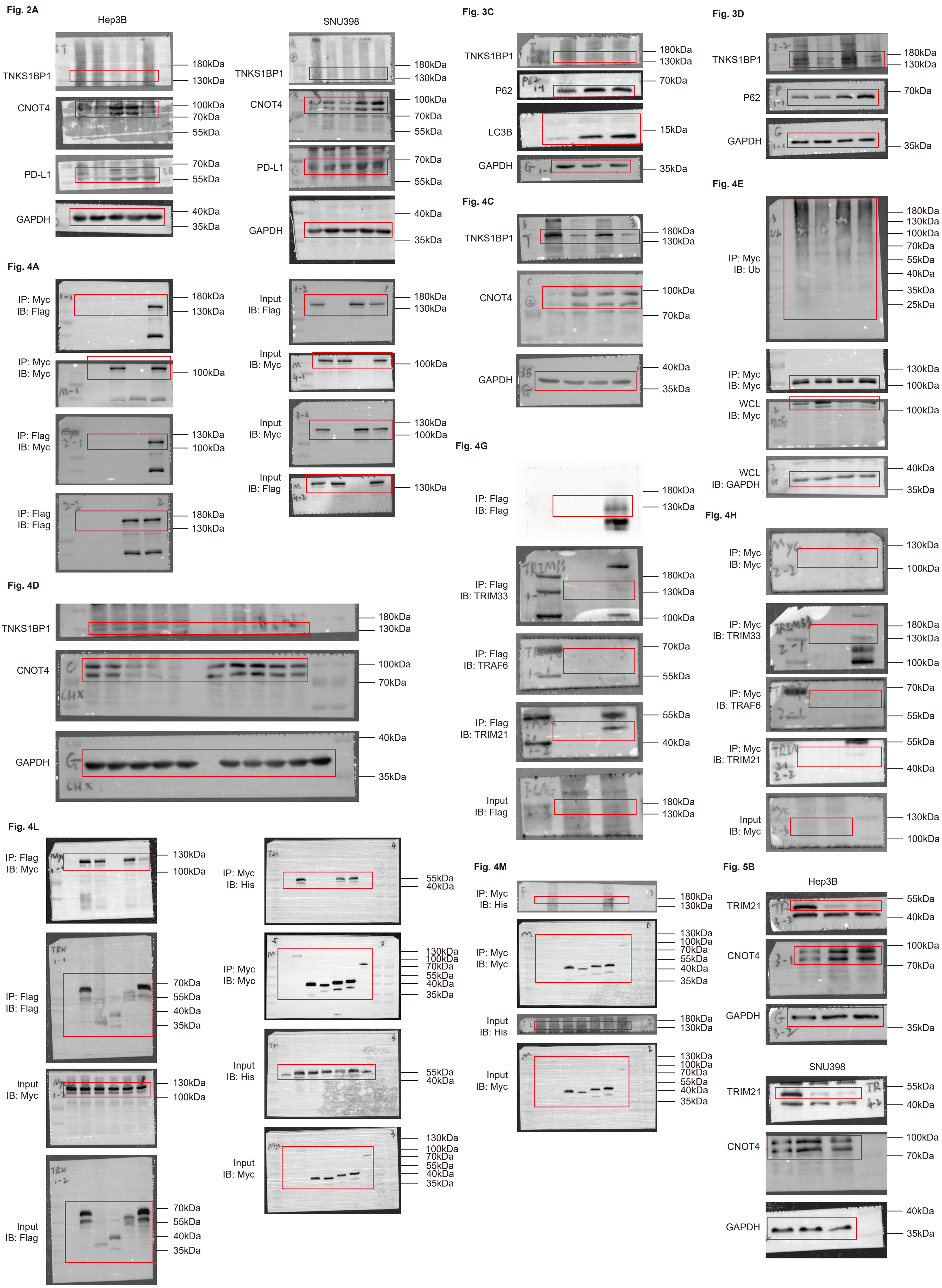

Fig. 5A

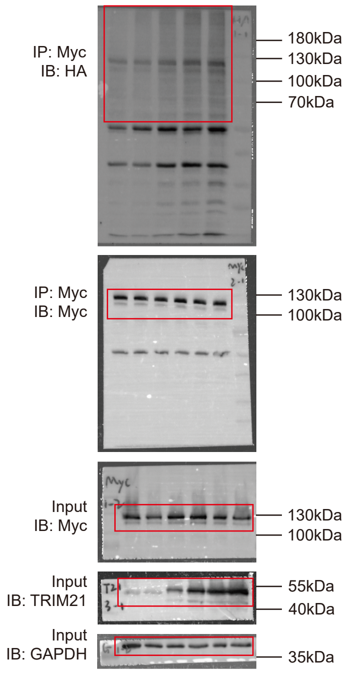

Fig. 5B

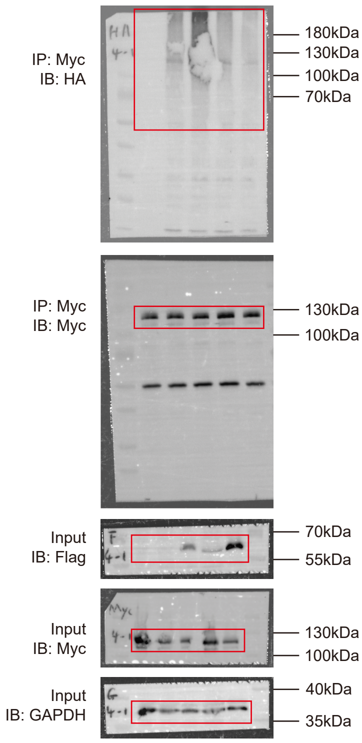

Fig. 5C

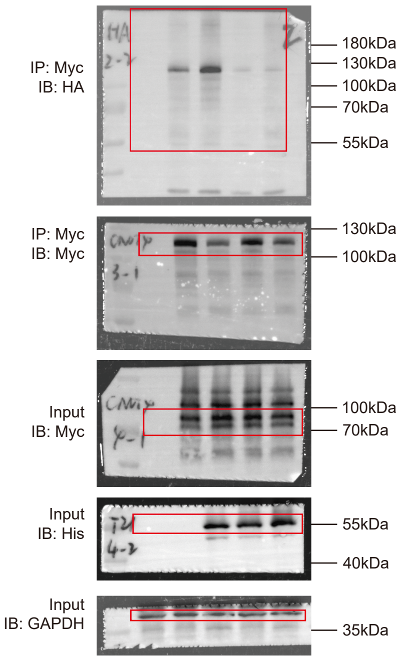

Fig. 5D

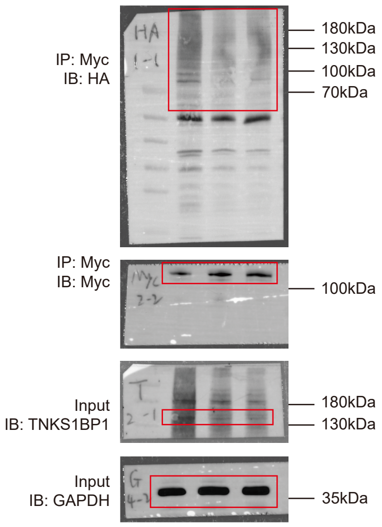

Fig. 5E

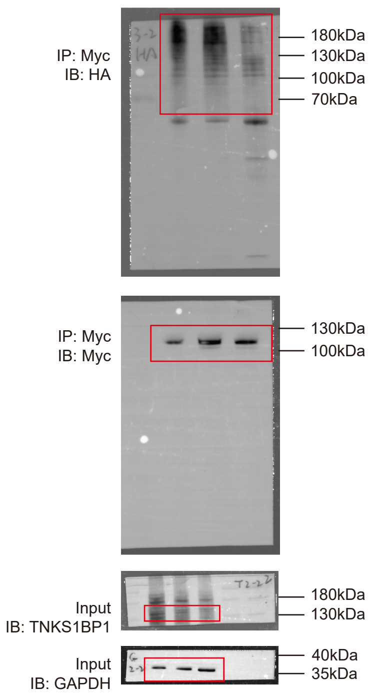

Fig. 5F

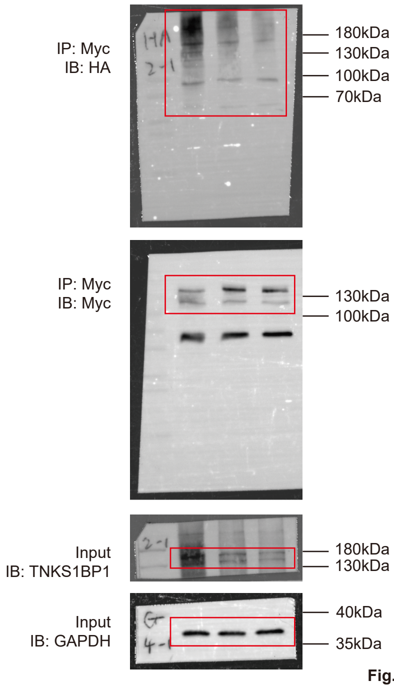

Fig. 5G

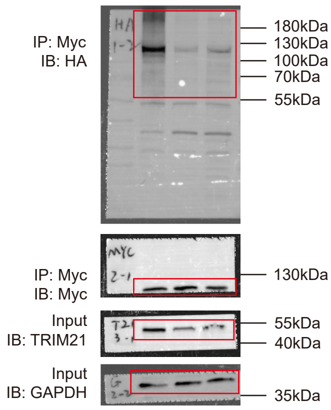

Fig. 5H

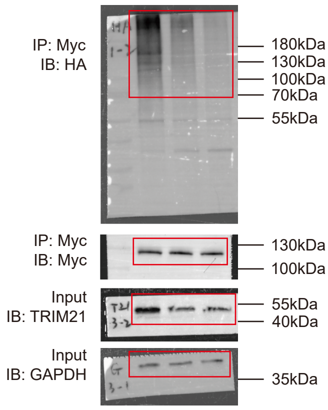

Fig. 5I

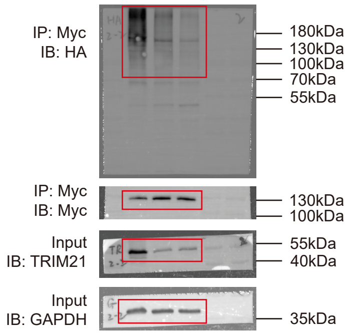

Fig. 5J

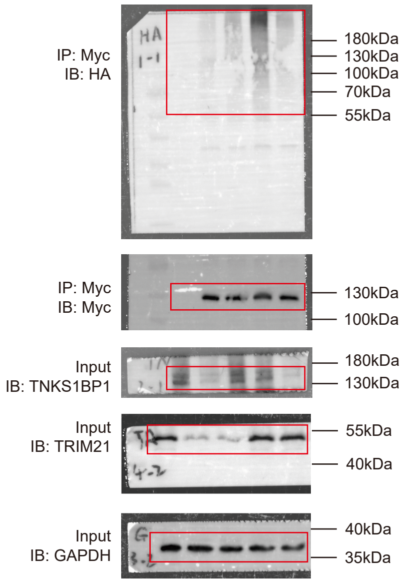

Fig. 5K

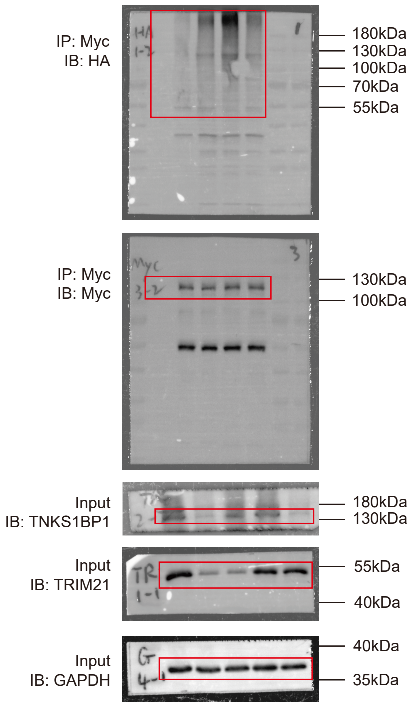

Fig. 5L

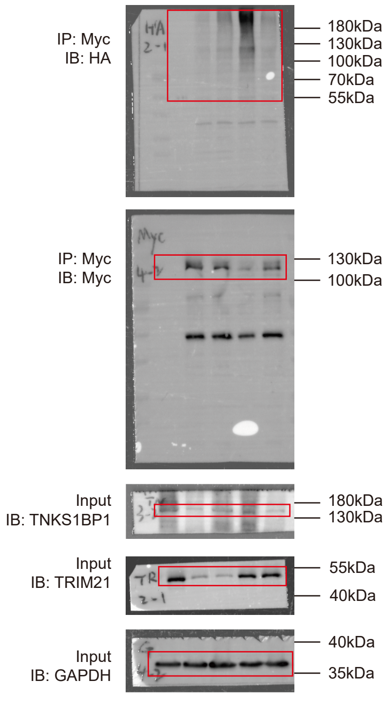

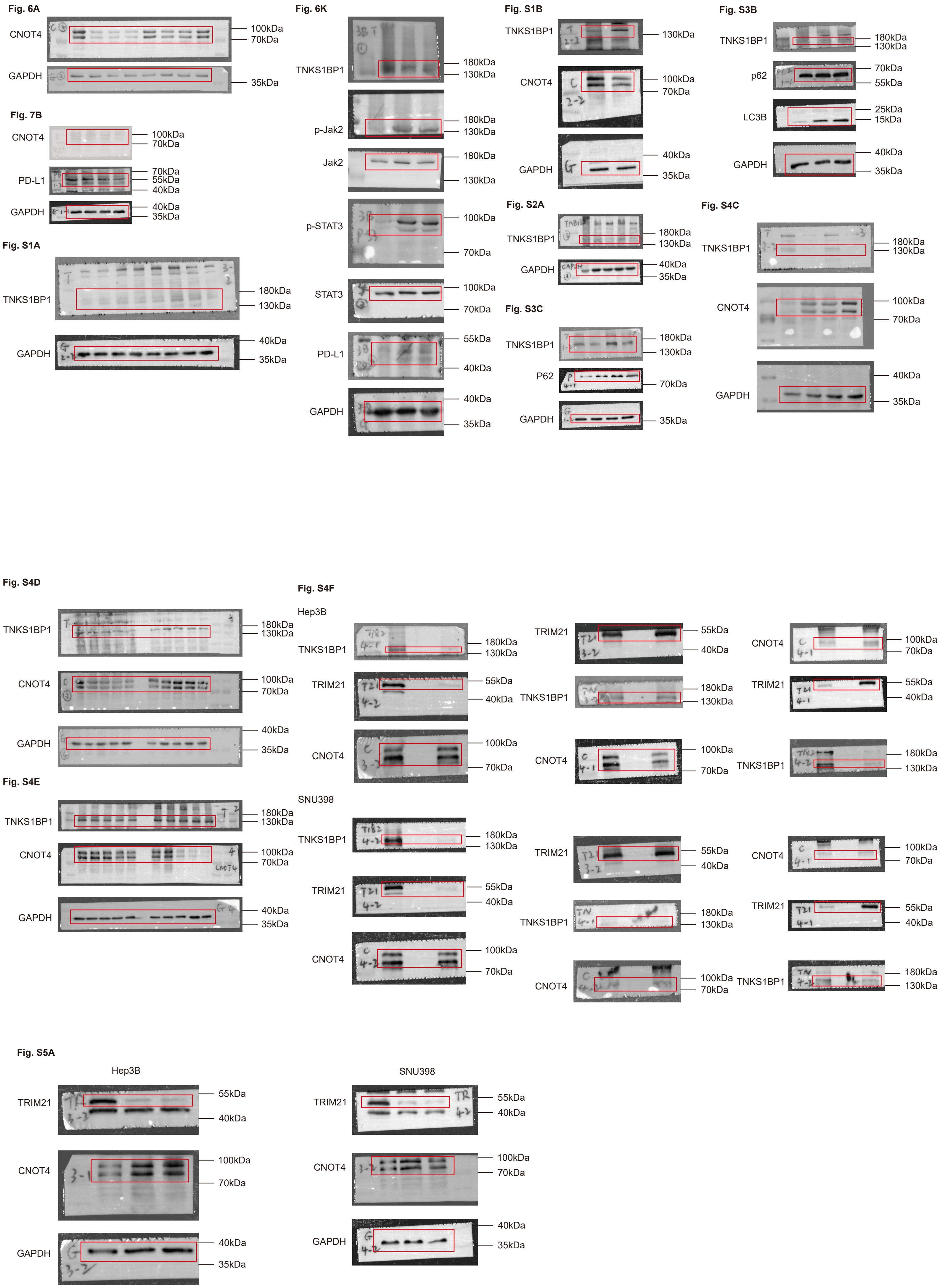
Fig. 6A
CNOT4
100kDa
70kDa
GAPDH
35kDa
Fig. 7B
CNOT4
100kDa
70kDa
PD-L1
70kDa
55kDa
40kDa
GAPDH
40kDa
35kDa
Fig. S1A
TNKS1BP1
180kDa
130kDa
GAPDH
40kDa
35kDa
Fig. 6K
TNKS1BP1
180kDa
130kDa
p-Jak2
180kDa
130kDa
Jak2
180kDa
130kDa
p-STAT3
100kDa
70kDa
STAT3
100kDa
70kDa
PD-L1
55kDa
40kDa
GAPDH
40kDa
35kDa
Fig. S1B
TNKS1BP1
130kDa
CNOT4
100kDa
70kDa
GAPDH
40kDa
35kDa
Fig. S2A
TNKS1BP1
180kDa
130kDa
GAPDH
40kDa
35kDa
Fig. S3C
TNKS1BP1
180kDa
130kDa
P62
70kDa
GAPDH
35kDa
Fig. S3B
TNKS1BP1
180kDa
130kDa
p62
70kDa
55kDa
LC3B
25kDa
15kDa
GAPDH
40kDa
35kDa
Fig. S4C
TNKS1BP1
180kDa
130kDa
CNOT4
100kDa
70kDa
GAPDH
40kDa
35kDa
Fig. S4D
TNKS1BP1
180kDa
130kDa
CNOT4
100kDa
70kDa
GAPDH
40kDa
35kDa
Fig. S4E
TNKS1BP1
180kDa
130kDa
CNOT4
100kDa
70kDa
GAPDH
40kDa
35kDa
Fig. S4F
Hep3B
TNKS1BP1
180kDa
130kDa
TRIM21
55kDa
40kDa
CNOT4
100kDa
70kDa
TRIM21
55kDa
40kDa
TNKS1BP1
180kDa
130kDa
CNOT4
100kDa
70kDa
CNOT4
100kDa
70kDa
TRIM21
55kDa
40kDa
TNKS1BP1
180kDa
130kDa
SNU398
TNKS1BP1
180kDa
130kDa
TRIM21
55kDa
40kDa
CNOT4
100kDa
70kDa
TRIM21
55kDa
40kDa
TNKS1BP1
180kDa
130kDa
CNOT4
100kDa
70kDa
CNOT4
100kDa
70kDa
TRIM21
55kDa
40kDa
TNKS1BP1
180kDa
130kDa
Fig. S5A
Hep3B
TRIM21
55kDa
40kDa
CNOT4
100kDa
70kDa
GAPDH
40kDa
35kDa
SNU398
TRIM21
55kDa
40kDa
CNOT4
100kDa
70kDa
GAPDH
40kDa
35kDa

Fig. S5B

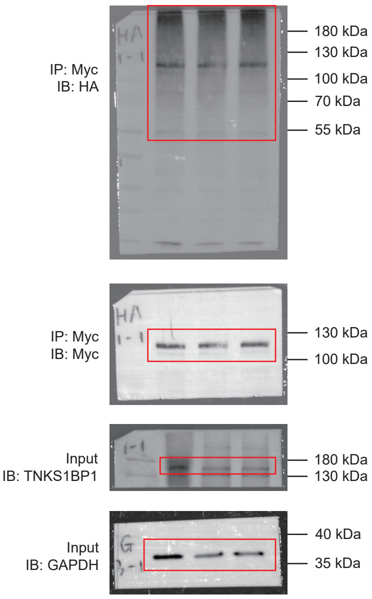

Fig. S5C

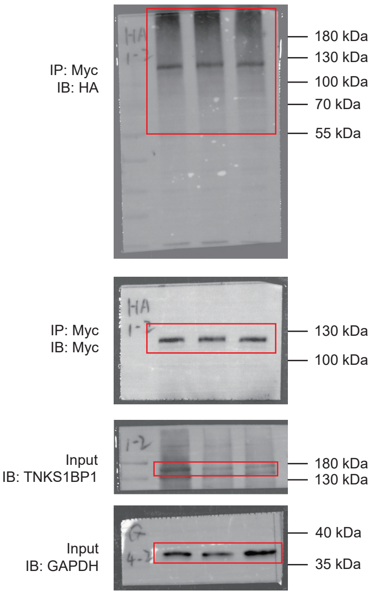

Fig. S5D

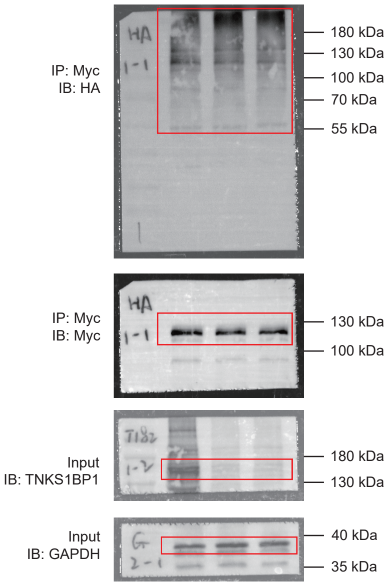

Fig. S5E

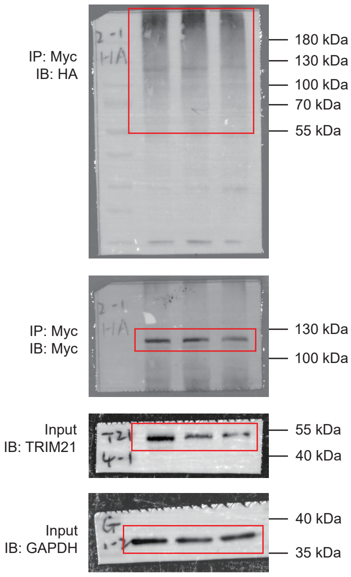

Fig. S5F

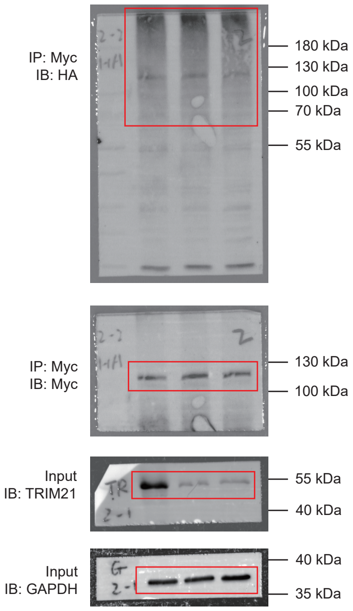

Fig. S5G

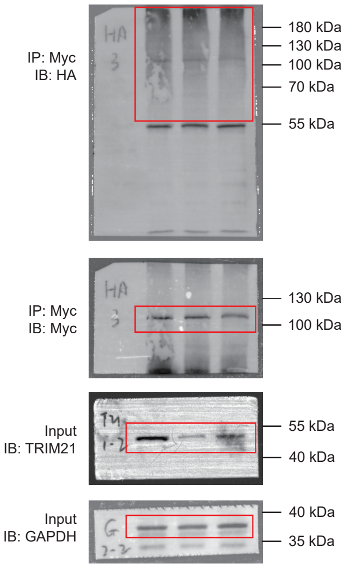

Fig. S5H

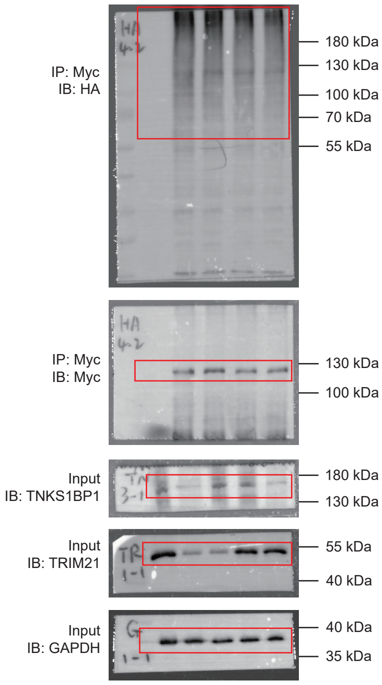

Fig. S5I

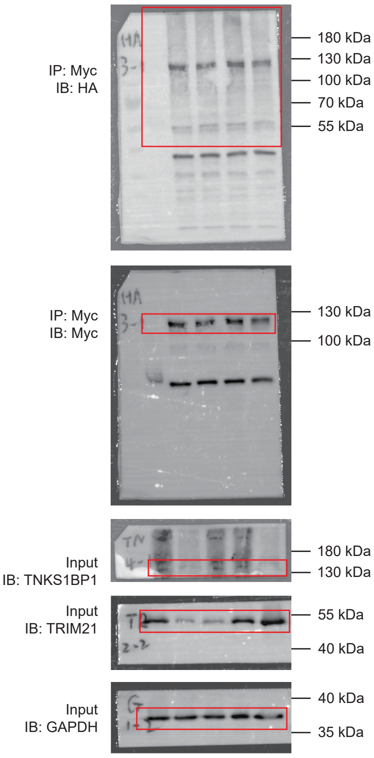

Fig. S5J

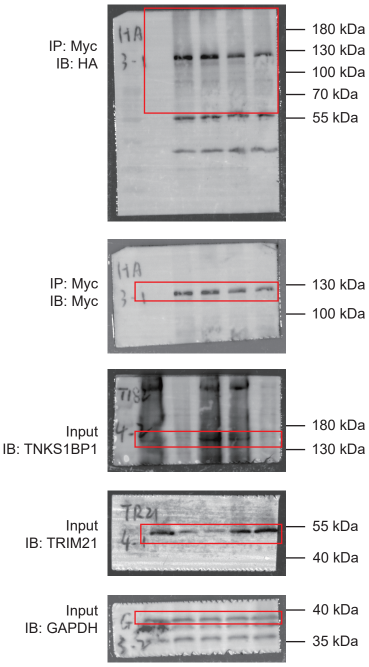

Fig. S6C

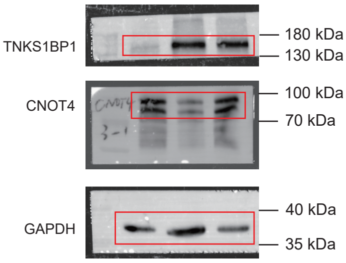

Fig. S6L

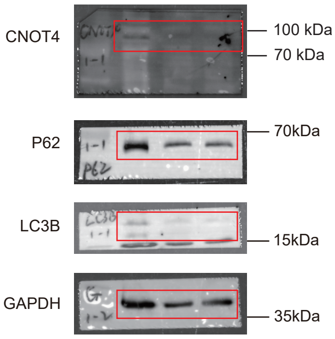

Fig. S6N

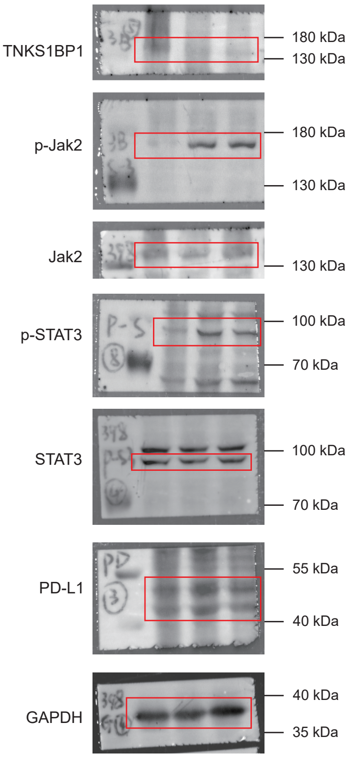

Fig. S6O

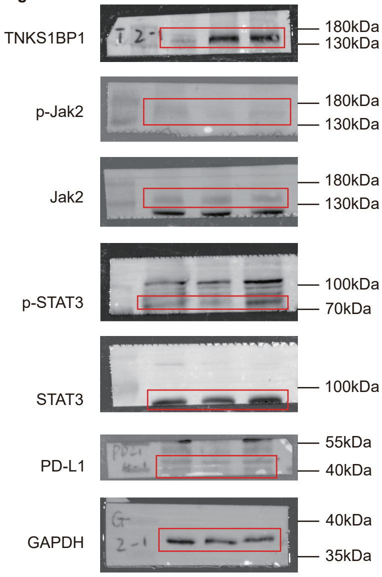

Supplement: Supplementary file 12 — Original Western Blots [file 41419_2024_6897_MOESM12_ESM.pdf]
